# Optimization of Lipid Nanoparticles with Robust Efficiency for the Delivery of Protein Therapeutics to Augment Cancer Immunotherapy

**DOI:** 10.1002/advs.202500844

**Published:** 2025-03-08

**Authors:** Lanfang Ren, Zeda Zhao, Yuqing Chao, Panting Yu, Zhoufang Mei, Bing Du, Yiyun Cheng

**Affiliations:** ^1^ Shanghai Frontiers Science Center of Genome Editing and Cell Therapy Shanghai Key Laboratory of Regulatory Biology School of Life Sciences East China Normal University Shanghai 200241 P. R. China; ^2^ Zhejiang Provincial Key Laboratory of Pancreatic Disease First Affiliated Hospital School of Medicine Zhejiang University Hangzhou 310006 P. R. China; ^3^ Department of Pulmonary and Critical Care Medicine Center of Community‐based Health Research Shanghai Fifth People's Hospital Fudan University Shanghai 200240 P. R. China; ^4^ Joint Center for Translational Medicine Shanghai Fifth People's Hospital Fudan University and School of Life Science East China Normal University Shanghai 200240 P. R. China

**Keywords:** cancer immunotherapy, IL‐10, lipid nanoparticles, protein delivery, protein therapeutics

## Abstract

Lipid nanoparticles (LNPs) have been successful in delivering nucleic acids like siRNA and mRNA, but face challenges in protein delivery due to limited protein encapsulation and endosome escape. In this study, a family of LNPs is developed with robust high efficiency in addressing the multiple barriers in cytosolic protein delivery by incorporating clinically approved ionizable lipids into traditional cationic LNPs. The combination of cationic and ionizable lipids enables efficient protein binding and endosomal escape. Optimized LNPs efficiently deliver various proteins, including antibodies, enzymes, toxins, and Cas9 into living cells with reserved functions. Moreover, the designed LNPs show high serum stability during protein delivery, and the serum albumin adsorbed on LNPs facilitates protein delivery via albumin receptor‐mediated endocytosis, enabling highly efficient protein delivery in vivo. The optimized LNPs successfully deliver therapeutic proteins such as saporin and interleukin‐10 (IL‐10) to inhibit tumor growth in several animal models. The IL‐10 loaded LNPs enhanced the proliferation and cytotoxicity of T cells and improved the antitumor effect of adoptive transferred OT‐1 CD8^+^ T cells to melanoma. This study expands the applications of LNPs for the delivery of biomacromolecules, and the developed LNP formulations have enormous potential for the delivery of protein therapeutics to treat various diseases.

## Graphical abstract

This study develops lipid nanoparticles (LNPs) combining cationic and ionizable lipids for efficient cytosolic protein delivery. Optimized LNPs deliver functional proteins, including antibodies, enzymes, and Cas9, with high serum stability and albumin‐mediated endocytosis. They enable therapeutic delivery of saporin and IL‐10, inhibiting tumor growth and enhancing T cell activity, showcasing potential for protein therapeutic applications in disease treatment.

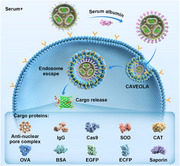

## 1 Introduction

Compared with small‐molecule drugs, protein‐based therapeutics have shown high potency and biosafety, and thus have been widely used in the treatment of diseases such as cancers, metabolic syndromes, and autoimmune diseases.^[^ ^1^ ^]^ However, protein drugs face various biological barriers, including a relatively short half‐life, vascular endothelial barriers, and limited cell and tissue penetration.^[^ ^2^ ^]^ Most of current protein‐based therapeutics are membrane impermeable, making it difficult to act on intracellular targets. Therefore, it is highly desired to develop efficient and robust delivery systems for protein drugs.

Various delivery systems have been developed to facilitate protein delivery, such as cell‐penetrating peptides,^[^ ^3^ ^]^ lipid nanoparticles (LNPs),^[^ ^4^, ^5^ ^]^ inorganic nanoparticles,^[^ ^6^ ^]^ nanogels,^[^ ^7^ ^]^ exosomes,^[^ ^8^ ^]^ and cationic polymers.^[^ ^9^, ^10^, ^11^, ^12^, ^13^ ^]^ Among them, LNPs have been extensively explored due to diverse lipid candidates, facile and uniform fabrication by microfluidics, good biocompatibility, and long blood circulation.^[^ ^14^, ^15^ ^]^ Several LNP‐based nanoformulations have been approved by the Food and Drug Administration (FDA) in recent years. For example, LNP‐based mRNA vaccines have been successfully used in the prevention of COVID‐19.^[^ ^16^ ^]^ However, LNPs for efficient protein delivery especially intracellular protein delivery remains challenging. Unlike nucleic acids with high density of negative charges, proteins possess distinct physiochemical properties, such as net charges, hydrophobicity, and molecular sizes, and structure conformations. Thus, cargo proteins usually have limited binding sites for LNP carriers. To address this issue, the proteins are usually modified with other ligands such as super‐negatively charged proteins,^[^ ^17^, ^18^, ^19^ ^]^ sulfonates,^[^ ^20^ ^]^ oligonucleotides,^[^ ^7^ ^]^ and cis‐aconite^[^ ^21^ ^]^ or phenylboronic acid^[^ ^22^ ^]^ to enhance their binding with lipid carriers. However, these modifications are associated with complicated synthesis and may impair protein functions. Besides protein binding, the efficacy of protein‐loaded LNPs also depends on several key parameters, such as serum stability, cellular uptake, and endosome escape.^[^ ^2^ ^]^ Therefore, cationic lipid libraries are usually used to screen efficient formulations,^[^ ^15^, ^17^, ^23^, ^24^ ^]^ and there is limited knowledge on the structure‐function relationships of lipid carriers for intracellular protein delivery.

**Scheme 1:**
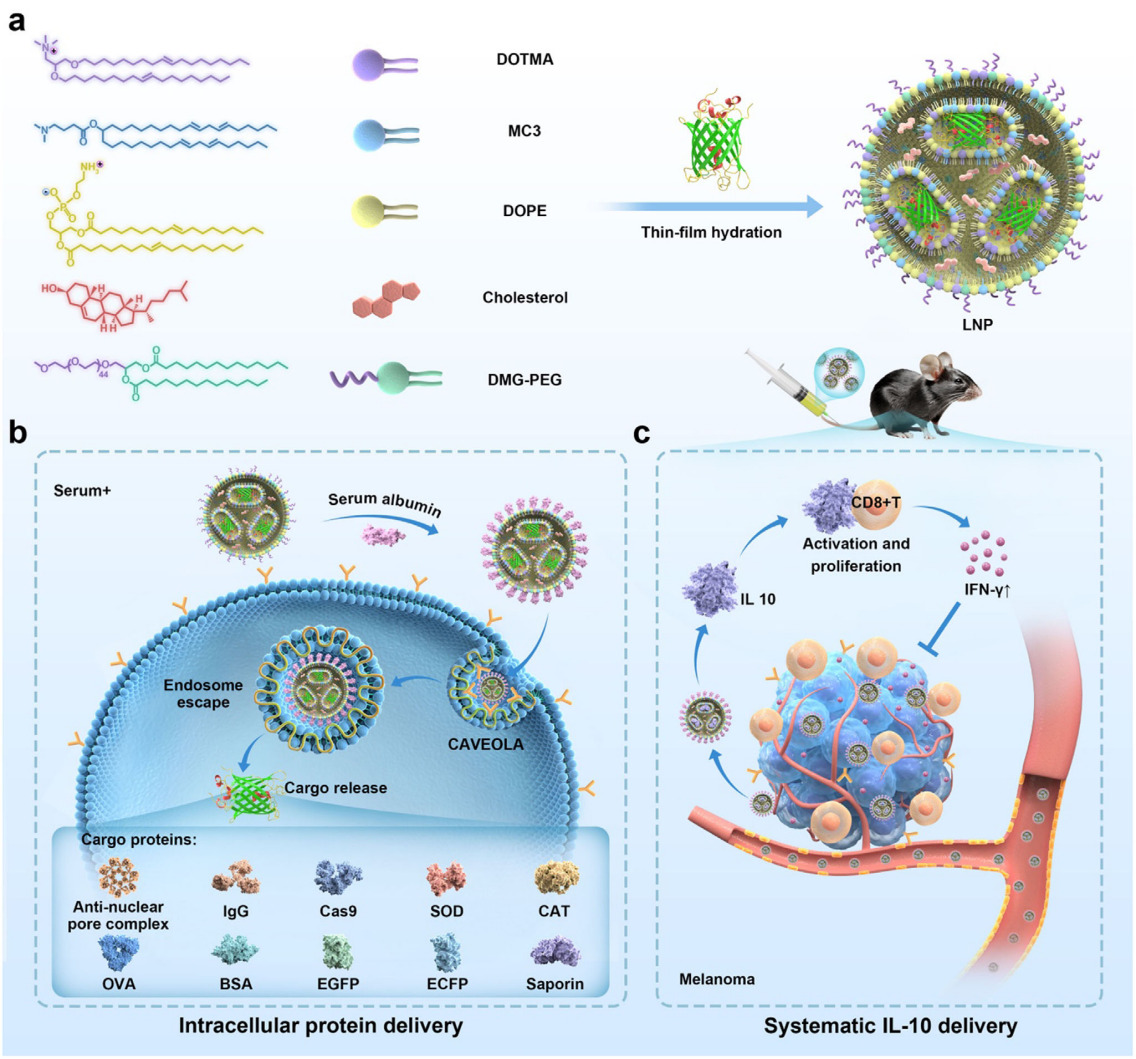
LNP nanoformulations for intracellular and in vivo protein delivery. a) Design of five‐component LNPs with high serum stability for efficient protein delivery. b) Intracellular trafficking of protein‐loaded LNPs. The LNPs showed robust efficacy in the delivery of various cargo proteins. c) Systematic administration of IL‐10 for enhanced adoptive transfer of OT‐1 CD8^+^ T cells to treat melanoma.

In this study, we rationally designed five‐component LNP formulations consisting of cationic lipid, ionizable lipid, polyethylene glycol (PEG)‐lipid, helper lipid, and cholesterol for efficient intracellular and in vivo protein delivery (**Scheme** **1**a). Cationic lipids such as 1,2‐dioleoyl‐3‐trimethylammonium‐propane (DOTAP) and 1,2‐di‐O‐octadecenyl‐3‐trimethylammonium propane (DOTMA) are beneficial for protein binding and endocytosis.^[^ ^25^, ^26^ ^]^ Ionizable lipids such as Dlin‐MC3‐DMA (MC3), ALC0315, SM102 play an import role in endosomal escape.^[^ ^27^, ^28^, ^29^ ^]^ The zwitterionic phospholipid (1,2‐dioleoyl‐sn‐glycero‐3‐phosphoethanolamine, DOPE) also facilitates endosomal membrane fusion and cargo release.^[^ ^30^ ^]^ In addition, the PEG‐lipid conjugate (1,2‐dimyristoyl‐sn‐glycerol‐methoxyPEG2000, DMG‐PEG) endows the co‐formulated LNPs with good serum tolerance due to the anti‐fouling properties of PEG on the nanoparticle surface. By balancing the ratio of cationic and ionizable lipids, we obtained highly efficient LNP formulations for cytosolic delivery of various cargo proteins including monoclonal antibodies, enzymes, toxins, and Cas9 ribonucleoprotein (RNP) into living cells. The protein‐loaded LNPs adsorb serum albumin on the surface and are efficiently internalized by treated cells via albumin receptor‐mediated and caveola‐dependent pathway, which enables efficient protein delivery in the presence of abundant serum proteins (Scheme 1b). Finally, the designed LNPs were used to deliver several cargo proteins such as toxin saporin and interleukin‐10 (IL‐10) to treat cancer. Chimeric antigen receptor T‐cells (CAR‐T) therapy has shown great success in the treatment of several hematopoietic malignancies. However, it has been less effective in the treatment of solid tumors due to T cell exhaustion and dysfunction.^[^ ^31^ ^]^ Recent studies have proved that cytokine IL‐10 can alleviate exhaustion, promote proliferation, and enhance the effector function of T cells, leading to the regression of solid tumors and metastatic cancers.^[^ ^32^, ^33^ ^]^ We found that IL‐10 loaded LNPs efficiently improve the efficacy of adoptive transferred OT‐1 CD8^+^ T cells to solid tumors, inhibiting tumor growth (Scheme 1c). These results prove that the designed LNPs are suitable for robust protein delivery and can be applied to multiple disease treatment scenarios.

## 2 Results and Discussion

### 2.1 Optimization of LNP Formulations for Intracellular Protein Delivery

**Figure 1:**
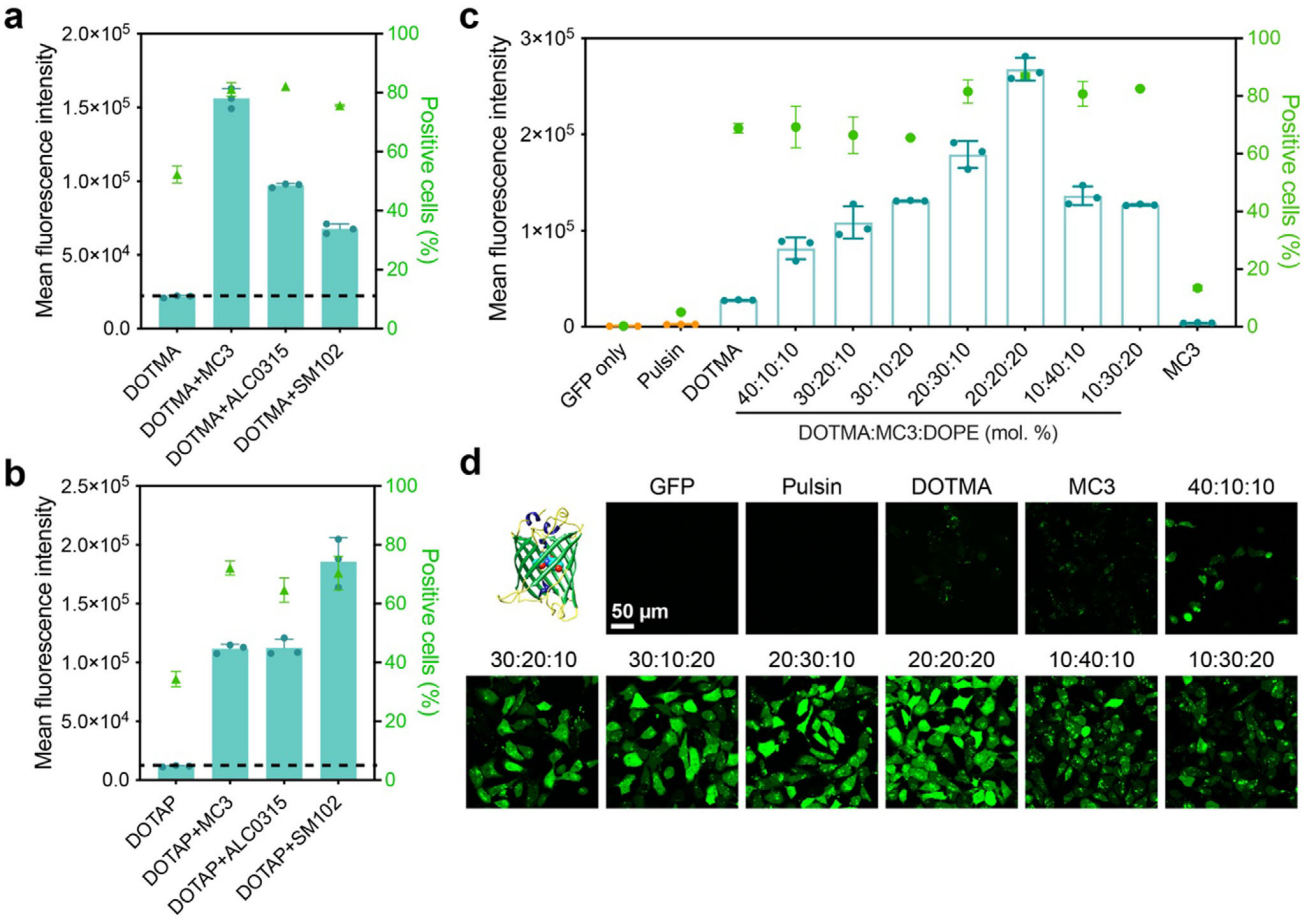
Optimization of LNP formulations for intracellular protein delivery. a,b) Mean fluorescence intensity of 143B cells treated with different LNP formulations. The concentration of GFP was fixed at 20 µg mL^−1^. c) Flow cytometry and d) confocal images of 143B cells treated with various LNP formulations for 16 h. GFP was visualized by green fluorescence.

To prove the important role of ionizable lipids in the LNP formulation, LNPs consisting of cationic lipid (DOTAP, DOTMA), ionizable lipids (MC3, ALC0315, SM102), helper lipid DOPE, DMG‐PEG, and cholesterol were prepared by a well‐established thin‐film hydration method. Cationic DOTAP and DOTMA LNPs (the molar ratio of cationic lipid/DOPE/cholesterol/DMG‐PEG is 50:10:38.5:1.5) were prepared as control materials. Green fluorescent protein (GFP) was used as the model protein. As shown in **Figure** **1**a,b, four‐component DOTAP or DOTMA LNPs showed relatively low efficacy in GFP delivery into osteosarcoma 143B cells. The addition of all the three types of ionizable lipids (the molar ratio of cationic lipid/ionizable lipid/DOPE/cholesterol/DMG‐PEG is 20:30:10:38.5:1.5) into the LNPs greatly increased the protein delivery efficacy. In addition, further regulating the incorporation ratio of ionizable lipid such as MC3 can result the better delivery (Figure S1, Supporting Information). This result is in line with a recent study by Alamgir et al., which demonstrated that the introduction of cationic lipid DOTAP into the conventional four‐component LNPs (consisting of MC3/distearoylphosphatidylcholine/cholesterol/DMG‐PEG) for siRNA delivery can promote the assembly of LNPs with anionically cloaked proteins via electrostatic actuation, and also showed better protein delivery compared to four‐component LNPs.^[^ ^20^ ^]^ In further studies, DOTMA and MC3 were chosen as the representative cationic and ionizable lipids, respectively to prepare the LNPs. The incorporation of MC3 did not increase the cytotoxicity of LNPs on the treated cells (Figure S2, Supporting Information). For nucleic acid delivery, acidic buffers were usually used to enhance the interactions between ionizable lipids and nucleic acids during LNPs formulation.^[^ ^34^ ^]^ However, acidic buffers may result in the denaturation of cargo proteins, and thus we used a neutral buffer (pH 7.4) during LNP formulation and protein loading. Compared to phosphate buffer saline (PBS) and the culture medium opti‐MEM, the 4‐hydroxyethyl piperazine ethanesulfonic acid buffer (HEPES), tris(hydroxymethyl)aminomethane (Tris), and acetate buffer showed higher efficacy for the LNPs during GFP delivery (Figure S3, Supporting Information). In the subsequent experiments, all the LNPs were prepared in the HEPES buffer (pH 7.4).

To further optimize efficacy of DOTMA/MC3 based LNPs, we prepared a series of LNPs by adjusting the molar ratios of DOTMA, MC3, and DOPE in the formulations (Table S1, Supporting Information). The contents of cholesterol and DMG‐PEG were fixed in all the prepared LNPs. As shown in Figure 1c, the GFP delivery efficacy of LNPs first increases with increasing MC3 contents in the formulations, and then decreases when excess MC3 is incorporated. LNP consisting of MC3, DOPE, DMG‐PEG, and cholesterol without DOTMA (simplified as MC3 in Figures) failed to deliver GFP into 143B cells. Among the investigated LNPs, the formulation prepared at DOTMA/MC3/DOPE molar ratio of 20:20:20 exhibited the highest performance in GFP delivery. The positive GFP cells delivered by the optimized LNP is more than 80%, and the delivery efficacy is much superior to commercial reagent Pulsin (Polyplus, France). In addition, the delivered GFP by the LNPs was observed via confocal imaging. After treating the cells for 1 h, we observed orange or yellow colocalized fluorescent spots in the cytoplasm or on the cell membrane, formed by the red fluorescence of LNPs labeled with DiI and the green fluorescence of the cargo protein GFP. After 6 h of treatment, the green fluorescence in the cytoplasm began to diffuse (Figure S4, Supporting Information), and the representative Pearson's colocalization coefficient (PCC) of fluorescence significantly decreased, indicating that the cargo protein was gradually released from the optimized LNPs (Figure S5, Supporting Information). After 16 h of treatment, the GFP was observed throughout the cytosol of 143B cells in Figure 1d, suggesting efficient protein release into the cytosols after delivery.

### 2.2 Mechanisms of LNPs in Protein Delivery

**Figure 2:**
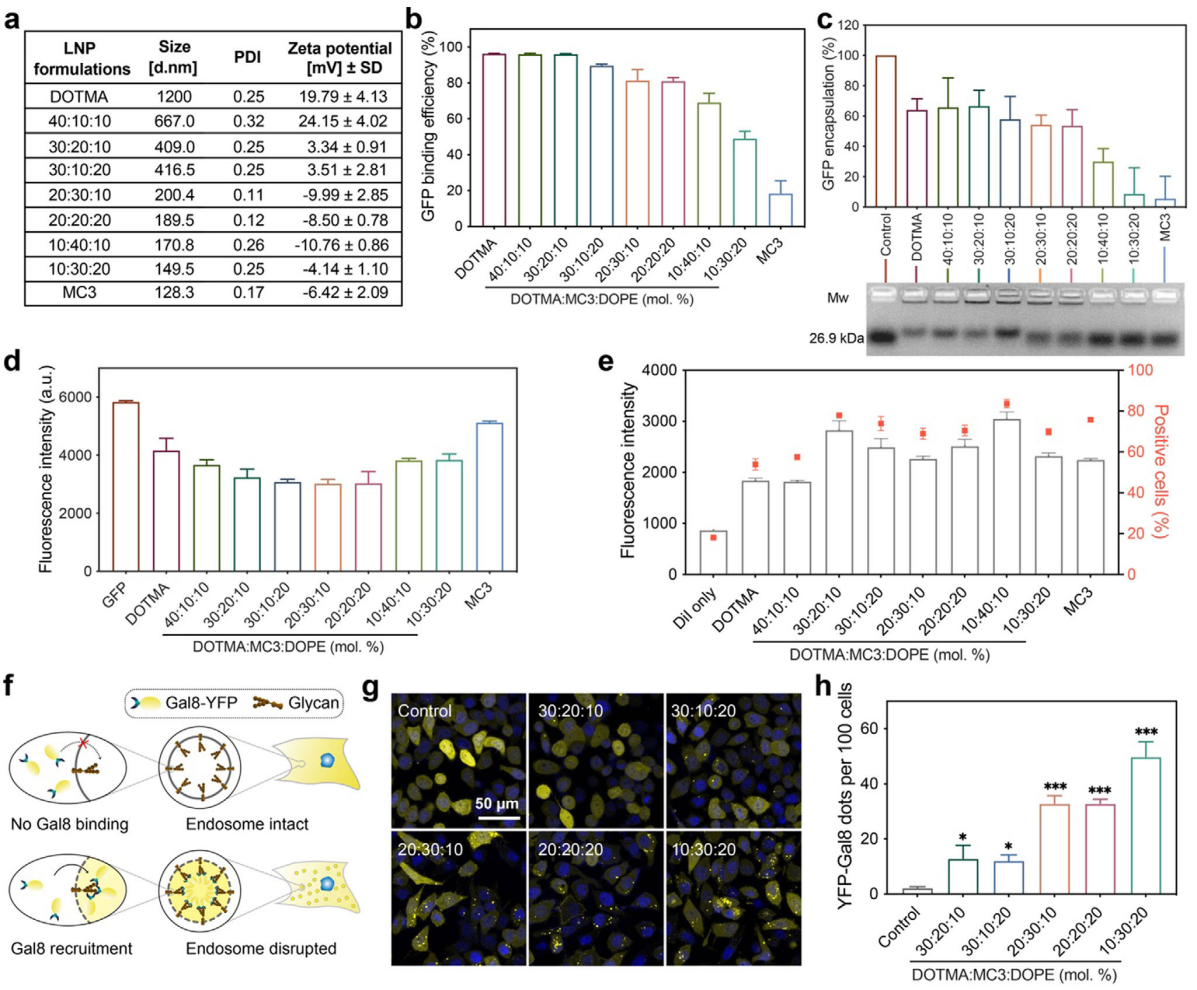
Characterization and mechanism of the LNPs during intracellular protein delivery. a) Hydrodynamic size and zeta potential of GFP‐loaded LNPs. b) GFP binding efficiency of LNPs in HEPES buffer via fluorescence spectroscopy. c) Agarose gel electrophoresis of GFP‐loaded LNPs. The encapsulation efficiency of GFP was measured according to the electrophoresis result. The total GFP for LNP encapsulation was used as a control. d) Peak fluorescence intensity of GFP‐loaded LNPs measured by fluorescence spectroscopy. The excitation wavelength was set at 450 nm, with free GFP serving as the control. e) Mean fluorescence intensity of cells treated with DiI‐labeled LNPs for 16 h. f) Schematic illustration of a Gal8 recruitment assay. g) Confocal images and h) number of fluorescence dots in YFP‐Gal8 expressing cells treated with different LNPs. YFP‐Gal8 was visualized by yellow fluorescence, while cell nuclei were stained with Hoechst 33342 and shown in blue fluorescence.

We further investigated the mechanisms of LNPs with different DOTMA/MC3/DOPE contents in GFP delivery. The zeta potential and hydrodynamic size of the LNPs were first characterized by dynamic light scattering (DLS, **Figure** **2**a). The DOTMA‐LNP without MC3 was positively charged due to the high contents of cationic lipids, and showed a relatively large hydrodynamic size above 1000 nm. The incorporation of MC3 gradually changed the LNPs from positively to negatively charged, and the nanoparticle size was decreased below 200 nm. Since efficient protein encapsulation is the prerequisite for intracellular protein delivery, we then measured the GFP binding efficiency of the prepared LNPs. As shown in Figure 2b, the GFP binding efficiency by LNPs can be precisely modulated by tuning the contents of different lipids. Higher contents of cationic DOTMA resulted in higher GFP binding efficiency, while the increase of MC3 and DOPE contents led to decreased binding efficiency. The isoelectric point (pI) of GFP is 6.2,^[^ ^35^ ^]^ and thus the protein is negatively charged in the neutral buffer. In this case, a higher content of cationic lipid DOTMA is beneficial for GFP binding via electrostatic interactions. The protein‐loaded LNPs were further analyzed using agarose gel electrophoresis. As shown in Figure 2c, only a small proportion of GFP remained unencapsulated by the LNPs. The increase in MC3 and DOPE contents resulted in a higher amount of unbound GFP under the electrophoresis condition. These results are in accordance with the protein binding efficiency of different LNPs. Besides, we evaluated the binding of cargo proteins with the lipid carriers using a fluorescence spectroscopy. As shown in Figure 2d, the loading of GFP in the LNPs led to efficient fluorescence quenching, suggesting interactions between the lipids and the cargo protein.^[^ ^36^ ^]^ Surprisingly, the most efficient GFP fluorescence quenching was achieved at DOTMA/MC3/DOPE ratios of 20:30:10 and 20:20:20. This result suggested that GFP molecules are bound more tightly with the carriers in these LNPs. Besides ionic interactions between DOTMA and GFP, hydrophobic interactions and hydrogen bonding between other lipids and GFP also play an important role in protein loading.^[^ ^34^ ^]^ We also tested the loading and delivery of LNPs with two other types of proteins: bovine serum albumin (BSA, pI 4.7, 69.3 kDa) and immunoglobulin G (IgG, pI 8.0, 150 kDa). We found that LNPs with different lipid ratios exhibited similar trends in loading and delivery efficiency for negatively charged proteins (Figure S6a,c, Supporting Information). For positively charged proteins, such as IgG, the loading efficiency was low in LNPs with a higher proportion of DOTMA due to electrostatic repulsion between the protein and the LNP. However, among the LNPs we screened, LNPs with a DOTMA/MC3/DOPE molar ratio of 20:20:20 exhibited better loading and delivery performance as well (Figure S6b,d, Supporting Information). The above results indicate that LNPs with proper physiochemical properties and high protein loading efficiency can be prepared by adjusting the ratio of lipids in the LNPs.

Next, we compared the cellular uptake of LNPs prepared at various DOTMA/MC3/DOPE ratios. A lipophilic dye 1,1′‐dioctadecyl‐3,3,3′,3′‐tetramethylindo‐carbocyanine perchlorate (DiI) was incorporated into the LNPs for intracellular tracking.^[^ ^37^ ^]^ As shown in Figure 2e, the LNPs prepared with DOTMA/MC3/DOPE ratios ranging from 30:20:10 to 10:30:20 demonstrated more efficient cellular internalization by the treated cells compared to other ratios. We further investigated the behaviors of prepared LNPs in endosomal escape, which is also a crucial parameter during intracellular delivery. A galectin 8 (Gal8) recruitment assay was used to evaluate the level of endosomal disruption by the LNPs. Gal8 is a cytoplasmic protein that can selectively bind with glycosylated moieties in the internal endosome membrane.^[^ ^38^ ^]^ Cells stably expressing Gal8‐yellow fluorescent protein (YFP) fusion protein have been widely used to evaluate endosomal escape. If the endosome is disrupted, the cytoplasmic Gal8‐YFP can be recruited to damaged endosomes, forming discrete yellow fluorescent spots,^[^ ^39^ ^]^ as depicted in Figure 2f. As shown in Figure 2g, LNPs with a higher DOTMA content resulted in fewer yellow fluorescent spots, while those with a higher proportion of ionizable lipid MC3 generated much more yellow spots in the treated cells (Figure 2h), suggesting that ionizable lipid MC3 in the LNPs can promote endosomal escape of the LNPs after endocytosis. According to the above results, we chose the LNP formulation prepared at 20:20:20 (simplified as LNP in later studies) as the lead LNP carrier in further studies.

**Figure 3:**
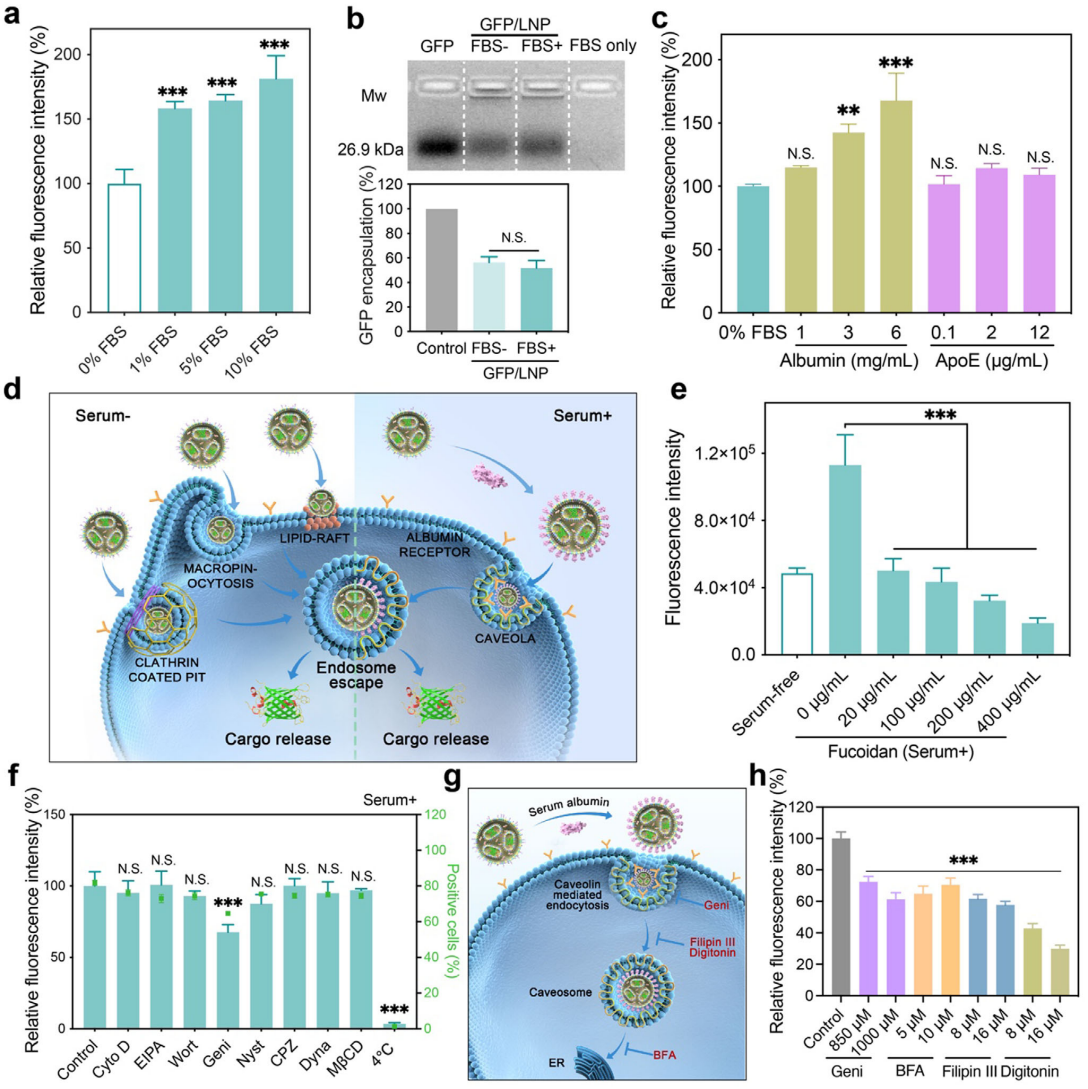
Serum tolerance and intracellular trafficking of LNPs. a) Relative fluorescence intensity of 143B cells treated with GFP‐loaded LNPs in serum‐free or serum‐contained medium. b) Agarose gel electrophoresis and percent of GFP encapsulated by LNPs in the presence and absence of FBS. The total GFP for LNP encapsulation was used as a control. c) Flow cytometry analysis of 143B cells treated with GFP‐loaded LNPs in medium containing albumin or ApoE. d) Schematic illustration of internalization pathways of LNPs in the absence and presence of serum proteins. e) Fluorescence intensity of cells treated with GFP‐loaded LNPs in the presence of fucoidan in serum‐containing medium. LNPs in serum‐free medium were used as a control. f) Relative fluorescence intensity of LNP‐treated cells pretreated with different endocytosis inhibitors in the presence of FBS. g) Schematic of caveola‐mediated intracellular trafficking for LNPs. h) Relative fluorescence intensity of cells pretreated with specific caveosome‐related inhibitors, followed by LNP treatment.

Another key challenge for nanocarriers in protein delivery especially for in vivo applications is their serum stability. Nanoparticles such as LNPs were adsorbed with serum proteins, and the proteins abundant in the blood may trigger the release of cargo proteins and affect the endocytosis as well as intracellular trafficking of the nanoparticles.^[^ ^40^, ^41^ ^]^ Therefore, we investigated the influence of fetal bovine serum (FBS) on the delivery efficacy of prepared LNPs. As shown in **Figure** **3**a, FBS addition greatly enhanced the GFP delivery efficacy of the LNPs on 143B cells. The presence of FBS did not result in the pre‐release of encapsulted GFP in the LNPs (Figure 3b). We further examined how serum proteins facilitate GFP delivery into the cells. Apolipoprotein E (ApoE), a reversible apolipoprotein, is mainly associated with lipid trafficking. After intravenous administration, serum ApoE usually binds onto LNPs, leading to liver accumulation of the nanoparticles.^[^ ^40^, ^42^, ^43^ ^]^ Albumin is the most abundant proteins in the serum and was reported to affect the endocytosis pathways and intracellular trafficking of nanoparticles.^[^ ^41^ ^]^ Previous studies have shown that albumin‐coated nanoparticles preferentially bind to the glycoprotein scavenger receptors gp30 and gp18 on the cell surface and promote endocytosis via caveola‐mediated or micropinocytosis‐related pathways.^[^ ^44^, ^45^ ^]^ We found that the GFP delivery efficacy of LNPs increases with increasing albumin concentrations, but is scarcely influenced by ApoE, suggesting that albumin plays a crucial role in LNPs mediated protein delivery in serum‐containing media (Figure 3c). The addition of fucoidan, a gp30 and gp18 inhibitor, dramatically inhibited the intracellular delivery of GFP by LNPs in a dose‐dependent manner, suggesting that endocytosis of GFP‐loaded LNPs depends on albumin receptors (Figure 3e). We further investigated the cell internalization pathways of GFP‐loaded LNPs using different endocytosis inhibitors in the presence and absence of 10% FBS. The LNPs were internalized through multiple pathways, such as macropinocytosis‐, lipid raft‐, and clathrin‐dependent endocytosis in serum‐free medium (Figure S7, Supporting Information). However, the internalization of nanoparticles in the serum‐containing medium was only inhibited by genistein (Geni), a caveola‐dependent endocytosis inhibitor (Figure 3d,f). Besides Geni, the uptake of GFP‐loaded LNP is also inhibited by filipin III (a cholesterol depletion agent), digitonin (a cholesterol depletion agent), and brefeldin A (BFA, a caveola‐disrupting agent and caveosome trafficking inhibitor). These results further proved that the albumin‐adsorbed LNPs were internalized via caveola‐mediated endocytosis (Figure 3g,h). Considering the high expression of albumin receptors on tumor cells,^[^ ^46^ ^]^ this property can be used for targeted protein delivery into tumor tissues.

### 2.3 Robustness of the LNPs in Protein Delivery

**Figure 4:**
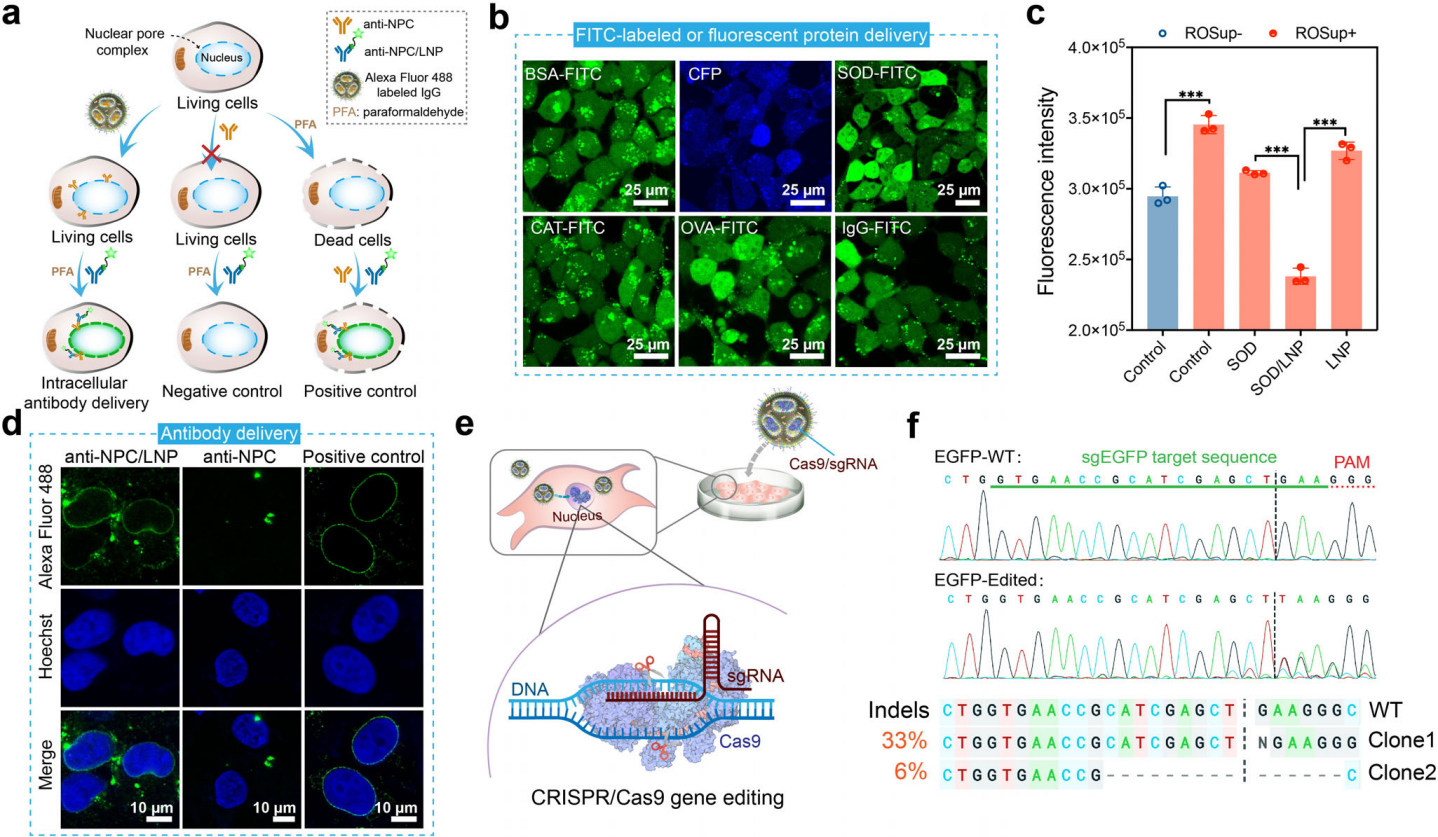
Robustness and versatility of LNPs in protein delivery. a) Schematic of intracellular antibody delivery and detection. b) Confocal images of 143B cells treated with LNPs loaded with different FITC‐labeled or fluorescent proteins. FITC and CFP were visualized by green and blue fluorescence, respectively. c) Intracellular ROS levels of LO2 cells treated with SOD/LNP, free SOD, and LNPs, respectively, followed by Rosup treatment. d) Immunofluorescence staining of 143B cells treated with LNPs loaded with anti‐NPC or free anti‐NPC for 16 h. Fixed cells treated with anti‐NPC and Alexa Fluor 488‐labeled secondary antibody were tested as a positive control. Alexa Fluor 488 was visualized by green fluorescence, while Hoechst‐stained nuclei were shown in blue fluorescence. e) Schematic of gene editing by LNPs loaded with Cas9 RNP. f) Sanger sequencing of the EGFP gene in 293T‐EGFP cells treated with LNP‐Cas9/sgEGFP.

We further examined whether the LNPs could achieve robust efficacy in the delivery of proteins with distinct properties. The molecular weight and isoelectric point of the cargo proteins, as well as the size, zeta potential, encapsulation efficiency, and loading efficiency of the LNPs encapsulating these proteins, are listed in Figure S8 (Supporting Information). Cargo proteins including BSA, superoxide dismutase (SOD), catalase (CAT), ovalbumin (OVA), and IgG were conjugated with fluorescein isothiocyanate (FITC) before intracellular delivery. As shown in **Figure** **4**b, all the FITC‐labeled proteins as well as cyan fluorescent protein (CFP) were successfully transported into the cytosol of 143B cells. We also investigated the versatility of LNPs in delivering monoclonal antibodies into living cells. The nuclear pore complex antibodies (anti‐NPC) that can specifically target the NPC on nucleus membrane were loaded in the LNPs. After intracellular delivery, the treated cells were fixed and stained by Alexa Fluor 488‐labeled secondary antibody to visualize the localization of anti‐NPC. As shown in Figure 4a,d, the 143B cells treated as described above showed obvious green fluorescence signals on the nucleus membrane, suggesting that anti‐NPC was efficiently delivered into the cells with maintained biological activity.

SOD, one of the antioxidant enzymes in cells, is responsible for catalyzing superoxide anions into hydrogen peroxide to protect the cells against excessive reactive oxygen species (ROS).^[^ ^47^ ^]^ As shown in Figure 4c, SOD could be efficiently delivered into human liver LO2 cells by the LNPs, and the delivered protein efficiently down‐regulated the ROS in the cells triggered by ROSup. We also evaluated the efficacy of LNPs in the delivery of Cas9 RNP into cells for CRISPR/Cas9 genome editing (Figure 4e). After PCR amplification and sequencing, we verified the typical insertions (Clone 1) and deletions (Clone 2) at the target sgEGFP sequence in comparison with the wild‐typed (WT) EGFP gene, suggesting the LNP efficiently delivered Cas9 RNP into 293T cells stably expressing EGFP (293T‐EGFP cells) and resulted in detectable gene editing activity (Figure 4f). These results demonstrated the robustness and versatility of the prepared LNPs in cytosolic protein delivery for various applications.

### 2.4 Saporin‐Loaded LNPs for Cancer Therapy

**Figure 5:**
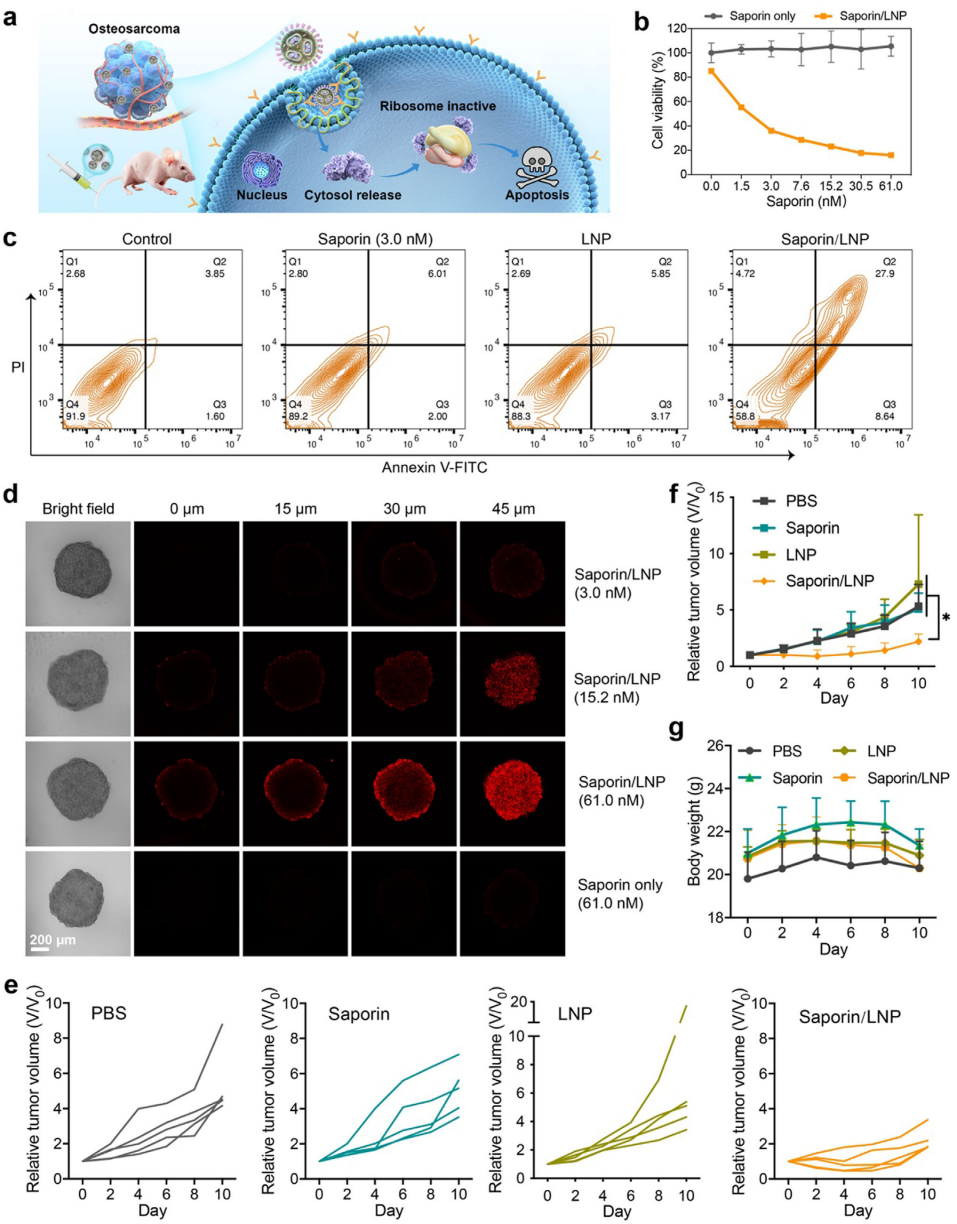
In vivo saporin delivery by LNPs for the treatment of osteosarcoma. a) Schematic illustration of intravenous saporin/LNP administration for cancer therapy. b) Viability of 143B cells treated with saporin/LNP at different saporin concentrations. c) Annexin V‐FITC and PI staining of 143B cells after treatment with different samples. The saporin concentration was fixed at 3.0 nm. d) Confocal scanning microscopy images of 143B spheres treated with different samples. Saporin was labeled with Cy5.5 (red fluorescence). These images were captured at 15 µm intervals from a fixed height on the spheroid toward its center. e) Tumor growth, f) relative tumor volume, and g) body weight of mice during the therapy (*n* = 5).

We then investigated the in vivo delivery efficacy of the LNPs using saporin as the model protein (**Figure** **5**a). Saporin, a type I ribosome‐inactivating protein, can depurinate the sarcin‐ricin loop from 28S subunit of ribosomes and irreversibly arrest protein translation, and induce cell death.^[^ ^48^ ^]^ However, the short plasma half‐life and membrane impermeability hinder its applications in clinical cancer therapy. We therefore prepared saporin‐loaded LNP and evaluated its therapeutic efficacy in vitro and in vivo. As shown in Figure 5b, incubation with free saporin or empty LNPs did not cause obvious toxicity to 143B cells, whereas LNPs loaded with saporin (referred to as saporin/LNP) showed dose‐dependent cytotoxicity on the cells. Annexin V‐FITC and propidium iodide (PI) staining of 143B cells showed that saporin/LNP induced significant apoptosis in the treated cells (Figure 5c). These results indicate that the designed LNPs can effectively deliver saporin into cancer cells with reserved bioactivity. Subsequently, we evaluated the tissue penetration ability of saporin/LNP in a 3D multicellular tumor spheroid model (Figure 5d). LNPs loaded with higher concentrations of Cyanine 5.5 (Cy5.5)‐labeled saporin resulted in efficient penetration into the tumor spheroids. In contrast, cells treated with free saporin exhibited minimal Cy5.5 fluorescent signals in the spheroid, indicating that the LNPs are capable of penetrating solid tissues during cancer therapy. Subsequently, we evaluated the in vivo therapeutic effect of saporin/LNP in mice bearing 143B osteosarcoma tumors. As shown in Figure 5e,f, intravenous administration of saporin/LNP significantly inhibited tumor growth, with therapeutic efficacy far superior to free saporin or empty LNPs. Furthermore, no significant changes in body weight were observed in treated mice compared to the PBS group (Figure 5g). Given the high cytotoxicity of saporin and the significant liver accumulation tendency of LNPs, it was essential to further investigate the biodistribution and safety of saporin/LNPs. To assess this, we studied the in vivo biodistribution of saporin‐loaded LNPs by labeling them with 1,1′‐dioctadecyl‐3,3,3′,3′‐tetramethylindotricarbocyanine iodide (DiR). As shown in Figure S9a (Supporting Information), DiR‐labeled LNPs, either alone or loaded with saporin, primarily accumulated in the liver, followed by the spleen. Additionally, DiR fluorescence was detected at the tumor site, indicating that LNPs also accumulated in the tumor (Figure S9b,c, Supporting Information). To evaluate safety, we measured serum ALT and AST levels, which serve as indicators of hepatic function. The treated groups showed no significant differences compared to the PBS group (Figure S9d,e, Supporting Information). Furthermore, histological analysis of major organs revealed no significant tissue damage (Figure S9f, Supporting Information). These results demonstrate that saponin‐loaded LNPs exhibit excellent therapeutic efficacy against tumors while causing no obvious adverse effects during treatment.

### 2.5 IL‐10 Loaded LNPs Augment Cancer Immunotherapy

**Figure 6:**
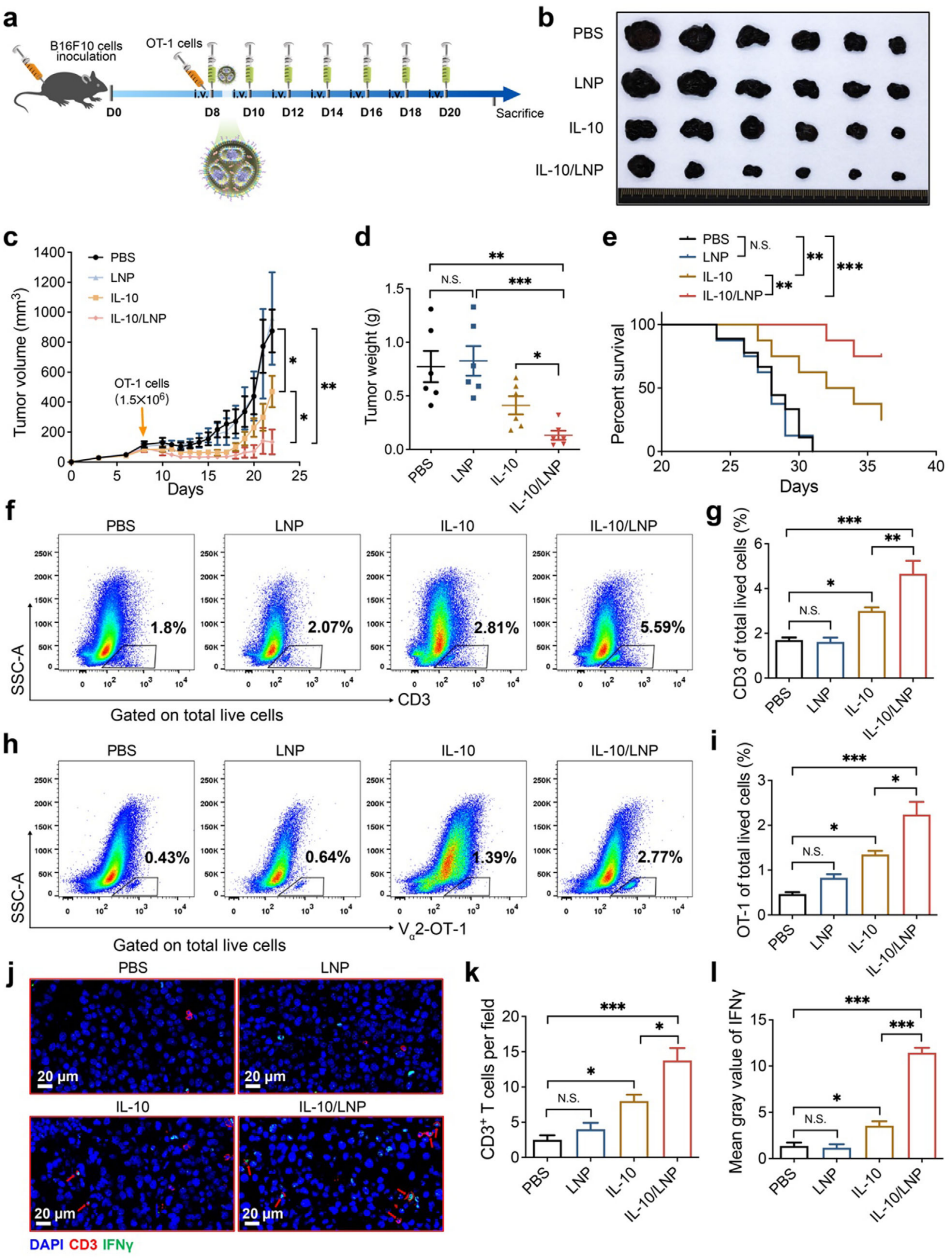
Combination of IL‐10/LNP and adoptive T cell transfer in cancer immunotherapy. a) Schematic of experimental timeline for melanoma treatment. b) Photographs of dissected B16F10‐OVA tumors on day 22 after tumor inoculation. c) Tumor growth and d) weights of B16F10‐OVA tumors (*n* = 6). e) Overall survival of B16F10‐OVA tumor‐bearing mice (log‐rank [Mantel–Cox] test; *n* ≥ 8). Representative gating strategy and percentages of f,g) tumor‐infiltrating CD3^+^ T cells and h,i) V_α_2‐OT‐1 T cells of total living cells in B16F10‐OVA tumor tissues on day 15 after tumor implantation analyzed by flow cytometry (*n* = 4). j) Representative images of multicolor immunofluorescence staining of CD3^+^ T cells and IFNγ in B16F10‐OVA mouse tumors. k) Quantification of CD3^+^ T cells and l) mean gray value of IFNγ in tumors (*n* = 4). The data are shown as mean ± SEM.

Cytokines perform many important functions in tumor immunotherapy. Previous studies have shown that IL‐10 can activate CD4^+^ and CD8^+^ cytotoxic T lymphocytes in vitro^[^ ^49^, ^50^ ^]^ and in vivo^[^ ^51^ ^]^ and promote the proliferation and cytotoxicity of CD8^+^ T cells in the tumor microenvironment. Recently, to extend the half‐life of IL‐10, IL‐10‐Fc fusion protein, and IL‐10‐expressing CAR‐T cells have been developed to alleviate CD8^+^ T cell exhaustion by reprograming T cell metabolic profiles and promoting the proliferation and effector function of CAR‐T cells, respectively.^[^ ^32^, ^33^ ^]^ Whereas, the releasing of IL‐10 by these auto‐expanded CAR‐T cells is unpredictable and has some safety risk especially in clinical. Accordingly, we investigated whether the designed LNPs could deliver IL‐10 to enhance the anti‐tumor effect of adoptive T cell transfer (ACT). As shown in Figure S10 (Supporting Information), the LNPs prolonged the half‐life of IL‐10 in blood. Specifically, we assessed the synergy of IL‐10 loaded LNP (IL‐10/LNP) with the adoptive transfer of OT‐1 CD8^+^ T cells using the poorly immunogenic and aggressive B16F10‐OVA melanoma model without lymphodepletion preconditioning. As the experimental timeline depicted, B16F10‐OVA cells were injected into C57BL/6 mice subcutaneously (10^6^ cells mouse^−1^). On day 8 after tumor inoculation, the mice received intravenous injections of activated OT‐1 CD8^+^ T cells (1.5 × 10^6^ cells mouse^−1^), followed by intravenous administration of PBS, LNPs, IL‐10, or IL‐10/LNP, respectively (**Figure** **6**a). While the administration of IL‐10 improved the efficacy of ACT, the combination of IL‐10/LNP with ACT resulted in a significantly greater reduction in both tumor size and weight (Figure 6b–d) and longer survival of tumor‐bearing mice (Figure 6e) than IL‐10/ACT and ACT alone. We then dissected B16F10 tumors to determine the IL‐10 levels in tumor tissues. The concentration of IL‐10 in tumors treated with intravenous IL‐10/LNP was higher than in those treated with free IL‐10 (Figure S11, Supporting Information). We also dissected B16F10 tumors to measure the tumor‐infiltrating OT‐1 CD8^+^ and CD3^+^ T cells on day 15 after tumor inoculation. Flow cytometry showed that these cells were more numerous in tumors treated with IL‐10/LNP and ACT than in the control groups (Figures 6f–i; S12, Supporting Information). Similarly, immunofluorescent staining showed a significantly higher number of CD3^+^ T cells in tumors treated with IL‐10/LNP and ACT than in other controls (Figure 6j,k). Most importantly, the expression of interferon‐γ (IFNγ) was significantly higher in the CD3^+^ T cells of mice treated with IL‐10/LNP and ACT (Figure 6l). These results suggest that IL‐10/LNP can enhance the antitumor effects of ACT by promoting the proliferation and cytotoxicity of T cells, which has broad clinical application in T cells mediated immune therapy.

## 3 Conclusion

In this study, we developed a type of LNPs consisting of cationic lipids and ionizable lipids that can effectively deliver various cargo proteins in vitro and in vivo. Mechanism studies revealed that fine‐tuning the ratio of cationic and ionizable lipids within the LNPs resolves the imbalance among protein binding, endosome escape, and intracellular release. The optimized LNPs not only retain the ability of cationic lipids to bind cargo proteins but also enhance endosomal escape and intracellular release through the incorporation of ionizable lipids. Moreover, the LNPs demonstrated excellent serum tolerance, and were internalized by cells via albumin receptors on the cell surface. Recognizing the distinct target specificities of protein therapeutics for intracellular versus extracellular targets, we systematically evaluated the in vivo delivery performance of the LNPs using two types of protein therapeutics. First, the LNPs exhibited robust protein delivery with preserved bioactivity, efficiently delivering the toxic protein saporin, which targets intracellular sites, to inhibit osteosarcoma growth. Additionally, cytokines have been well conceptualized as powerful tools in anticancer therapy for decades, yet only two (IFN‐α and IL‐2) have received FDA approval to date. The reasons are complex; however, short persistence and low efficacy remain the most critical challenges. In this study, we demonstrated that our LNPs not only prolong the blood half‐life of the cytokine IL‐10, but also enrich IL‐10 in tumor tissues which is essential for improving the efficacy of adoptive T cell therapy as well as immune checkpoint blockade drugs. We believe that with the help of our LNPs, the clinical efficacy and indications of cytokines could be expanded significantly. These LNPs can be further developed to treat various diseases by tailoring the encapsulated proteins and nanoparticle surface chemistry.

## 4 Experimental Section

### Materials

DOTMA, DMG‐PEG, and cholesterol were purchased from AVT Pharmaceutical Tech. (Shanghai, China). DOTAP, DOPE, MC3, SM102, and ALC0315 were purchased from Sinopeg Biotechnology (Xiamen, China). IgG, FITC, BSA, OVA, CAT, saporin, ApoE, Fucoidan, Cytochalasin D (Cyto D), Ethylisopropylamiloride (EIPA), Wortmannin (Wort), Geni, Nystatin (Nyst), Chlorpromazine (CPZ), Dynasore (Dyna) and Methyl‐β‐cyclodextrin (MβCD) were purchased from Sigma–Aldrich (St. Louis, USA). Cas9 protein and sgEGFP were purchased from GenScript (Nanjing, China). SOD, filipin III, digitonin, and BFA were obtained from Yuanye Bio. (Shanghai, China). Hoechst 33342 and DiI were purchased from Beyotime Biotechnology (Jiangsu, China). DiR were purchased from Energy Chemical (Anhui, China). Cy5.5 monosuccinimidyl ester was purchased from Meilunbio (Liaoning, China). Mouse recombinant IL‐10 was purchased from Absin (Shanghai, China). Pulsin was obtained from Polyplus‐transfection (Strasbourg, France). Enhanced green fluorescent protein (GFP) were expressed and purified in the lab.

### Synthesis of FITC‐Labeled Proteins

The proteins were dissolved in a PBS solution (pH 7.4). FITC dissolved in DMSO was added to protein solutions at a FITC/protein molar ratio of 3:1. The reaction mixture was stirred in the dark at room temperature for 24 h. After intensive dialysis against PBS and deionized water, the samples were freeze‐dried and stored at −20 °C for subsequent use.

### Preparation and Characterization of Protein‐Loaded LNPs

LNPs were prepared using the thin‐film hydration method. Generally, cationic lipid (DOTMA or DOTAP), ionizable lipid (MC3, SM102 or ALC0315), and helper lipids (DOPE, cholesterol, and DMG‐PEG) were dissolved in ethanol at a concentration of 10 mg mL^−1^. The cargo proteins were diluted with HEPES (20 mm). After mixing the lipids at different molar ratios and evaporating the organic solvent using a rotary evaporator RV8 (IKA, Staufen, Germany), the protein solutions were added to hydrate the thin lipid film. Sonication was carried out to prepare the final LNP formulations using a Sonics ultrasonic homogenizer (VCX150, Sonics & Materials, Inc., Connecticut, USA). To study the effect of solvents on LNP preparation and protein delivery efficiency, the cargo protein was also dissolved in PBS, optiMEM (Gibco, Thermo Fisher Scientific Inc., Massachusetts, USA), Tris (10 mm), and 10 mm acetate solutions, respectively before preparing the LNPs. DiI‐labeled LNPs were prepared similarly, with DiI dissolved in ethanol added to the lipid solutions at a mass ratio of 1:100, followed by thin‐film hydration.

The size and zeta potential of the LNP formulations were measured by dynamic light scattering using Zetasizer Nano ZS 90 (Malvern Instruments, Malvern, UK). GFP‐loaded LNPs (GFP/LNP) were characterized by fluorescence spectrophotometer F‐4500 (Hitachi Ltd., Tokyo, Japan). To quantify GFP binding efficiency, GFP‐loaded LNPs (16 µm for LNPs and 2.5 µg mL^−1^ for GFP) were centrifuged at 13000 rpm for 20 min. The supernatant was collected to measure the unbound GFP content using fluorescence spectroscopy. A standard curve of GFP in HEPES buffer was established (Y = 568.1^*^ X‐177.5, R^2^ = 0.999; where Y represents the fluorescence intensity and X represents the concentration of GFP in µg mL^−1^).

Agarose gel has a network structure. Under the influence of an electric field, free proteins can move toward the positive electrode in a solution with a pH above their isoelectric point, while the proteins loaded by LNPs will be trapped in the pores and unable to migrate. Therefore, agarose gel electrophoresis was performed to observe and quantify the protein encapsulation efficiency of LNPs in buffer solutions with or without 10% serum. Briefly, the prepared LNP formulations were supplemented with a glycerol‐water solution (50%, v/v) at a final volume ratio of 10:1. Subsequently, the LNP samples were loaded into wells of a 2% (w/v) agarose gel in 1× TAE buffer and subjected to electrophoresis at 90 V for 20 min, followed by imaging using a Tanon 2500 gel image system (Tanon, Shanghai, China). The protein encapsulation percentage of LNPs was determined through grayscale analysis of electrophoresis bands using Image J (National Institutes of Health, USA). The amount of encapsulated protein was calculated by subtracting the unencapsulated protein from the total planned protein loading amount, with the free protein from the planned loading serving as the control. Sodium dodecyl sulfate‐polyacrylamide gel electrophoresis (SDS‐PAGE) separates proteins solely based on their molecular weight and is independent of their isoelectric point and charge properties. Therefore, for proteins with higher isoelectric points (including IgG, IL‐10, Saporin, Cas9, and anti‐NPC), SDS‐PAGE was utilized to assess their loading efficiency. Briefly, LNPs loaded with proteins greater than 30 kDa were centrifuged at 13000 rpm for 20 min to remove the supernatant. LNPs loaded with proteins smaller than 30 kDa were filtered using ultrafiltration tubes with a molecular weight cutoff of 100 kDa at 6000 rpm for 60 min. The pellet or filtrate was resuspended in 1x protein loading buffer containing β‐mercaptoethanol (Epizyme Biotech, Shanghai, China) and denatured by heating at 100 °C for 10 min. A 10% or 15% polyacrylamide gel was prepared based on the molecular weight of the proteins, and the samples were loaded accordingly. Electrophoresis was performed at 80 V for the spacer gel and 120 V for the separation gel until the bromophenol blue reached the bottom of the gel. The gel was stained with Coomassie brilliant blue rapid staining solution (Epizyme Biotech, Shanghai, China). Finally, the gel was imaged using the Chemidoc MP imaging system (Bio‐Rad, California, USA).

### Cell Culture and In Vitro Protein Delivery

143B cells, LO2 cells, 293T‐EGFP cells and HeLa cells (stably expressing Gal8‐YFP) were cultured at DMEM (Gibco) supplemented with 10% fetal bovine serum (FBS) and 1% penicillin‐streptomycin. Unless otherwise stated, all cell culture and protein delivery experiments were performed in complete media. For in vitro protein delivery, 143B cells were seeded in 48‐well culture plates and incubated overnight. At 70% confluence, the cells were treated with LNP formulations diluted in complete media for 16 h. The working concentration of all LNPs for intracellular protein delivery are listed in Table S1 (Supporting Information). The concentration of model protein GFP, BSA‐FITC or IgG‐FITC was fixed at 20 µg mL^−1^. The treated cells were then washed with PBS and 0.04% (w/w) trypan blue solution to eliminate extracellular GFP fluorescence interference, and analyzed by flow cytometry using Cytoflex (Beckman Coulter, California, USA) or FACSCelesta (BD, New Jersey, USA). For confocal imaging, 143B cells were pre‐seeded in confocal dishes until they reached 70% confluence, and then incubated with GFP‐loaded LNPs in complete media at the same concentrations used in the 48‐well plates for 16 h. Afterward, the cells were rinsed with PBS and trypan blue solution, and photographed by laser scanning confocal microscopy (LSCM, Leica SP8, Leica Microsystems, Wetzlar, Germany) with excitation and emission wavelengths of 488 nm and a range of 500–550 nm, respectively.

To investigate the robustness of LNPs in cytosolic protein delivery, 14.4 µg mL^−1^ of anti‐NPC, FITC‐labeled proteins (20 µg mL^−1^ of IgG, 20 µg mL^−1^ of BSA, 20 µg mL^−1^ of SOD, 24 µg mL^−1^ of CAT, 20 µg mL^−1^ of OVA) and 20 µg mL^−1^ of fluorescent protein CFP were respectively encapsulated by 64 µM of LNPs, and then incubated with 143B cells as described above. The treated cells were imaged by LSCM (Ex: 488 nm and Em: 500–550 nm for FITC, while Ex: 405 nm and Em: 450–490 nm for CFP). For anti‐NPC delivery, the treated cells were fixed with 4% paraformaldehyde for 20 min, permeabilized with 0.2% Triton for 5 min, and blocked with 2% BSA for 30 min at room temperature. Cells treated with anti‐NPC served as a negative control. Cells fixed with 4% paraformaldehyde, treated with 0.2% Triton, and incubated with anti‐NPC served as a positive control. After staining with Hoechst 33342 for 10 min and Alexa Fluor 488‐labeled goat anti‐mouse secondary antibody (1:2000) for 1 h at 37 °C, the cells were imaged by LSCM (Ex: 405 nm and Em: 417–477 nm for Hoechst 33342, while Ex: 488 nm, Em: 500–550 nm for Fluor 488). The SOD enzyme activity in the treated cells was measured using a ROS detection kit (Beyotime Biotechnology, Jiangsu, China). Generally, LO2 cells were pre‐seeded in 48‐well plates until they reached 70% confluence. The cells were then incubated with free SOD, LNP or SOD/LNP for 16 h. The working concentration of SOD and LNP was fixed at 10 µg mL^−1^ and 64 µm, respectively. After that, all cells were replaced with fresh medium and treated with ROSup (1:500 diluted in serum‐free medium) for 1 hour to increase intracellular ROS, followed by DCFH‐DA treatment at a concentration of 10 µm for 20 min. Cells directly treated with DCFH‐DA were tested as a negative control. The fluorescence intensity of treated LO2 cells was measured by flow cytometry.

### Cellular Uptake and Intracellular Trafficking of LNPs

To examine the cellular uptake mechanism, 143B cells were pre‐seeded in 48‐well plates overnight until they reached 70% confluence. The cells were then treated with various inhibitors: Cyto D (10 µm, an inhibitor of macropinocytosis), EIPA (50 µm, an inhibitor of macropinocytosis), Wort (100 nm, an inhibitor of macropinocytosis), Geni (700 µm, an inhibitor of caveolae‐mediated endocytosis), Nyst (25 µg mL^−1^, an inhibitor of caveolae‐mediated endocytosis), CPZ (20 µm, an inhibitor of clathrin‐mediated endocytosis), Dyna (20 µm, an inhibitor of clathrin‐mediated endocytosis) and MβCD (5 mm, an inhibitor of lipid raft‐mediated endocytosis), respectively, for 1 h. Then the cells were incubated with GFP/LNP for another 16 h at 37 °C. GFP/LNP‐treated 143B cells incubated at 4 °C were used as an additional control. Inhibitors of caveolin‐dependent uptake pathway, including BFA, Filipin III, and Digitonin, were also pre‐treated with 143B cells for 1 hour, followed by LNP treatment as described above. To verify whether albumin receptor‐mediated endocytosis occurred, cells were co‐incubated with GFP/LNP and the albumin‐receptor inhibitor fucoidan. Cells incubated with GFP/LNP at 37 °C for 16 h without any inhibitor were used as controls (fluorescence intensity set to 100%). To investigate the effect of serum on intracellular protein delivery of LNPs, cells were treated with GFP‐loaded LNPs in the absence or presence of FBS. Albumin and ApoE, as the most common proteins adsorbed on LNPs, were added to fresh culture medium and incubated with GFP/LNP‐treated cells. These above‐treated cells were measured by flow cytometry.

To verify the cytosolic release of protein from LNPs, the GFP‐loaded LNPs were incorporated with hydrophobic DiI. 143B cells were pre‐seeded in confocal plates for 70% confluence, and then treated with DiI‐labeled LNPs in complete medium for 1 hour or 6 h. Subsequently, the colocalization of DiI and GFP was observed via LSCM (Ex: 488 nm and Em: 500–550 nm for GFP, while Ex: 552 nm and Em: 565–605 nm for DiI) and analyzed the PCC by ImageJ. PCC > 0.5 indicates colocalization, while PCC < 0.5 indicates no colocalization. The working concentration of GFP and LNPs were fixed at 20 µg mL^−1^ and 64 µm, respectively.

To evaluate the endosome escape capability of different LNPs, HeLa cells expressing Gal8‐YFP were cultured in confocal dishes and incubated in complete DMEM medium overnight. After 16 h of treatment with BSA‐loaded LNPs (20 µg mL^−1^ for BSA and 64 µm for LNP) in complete medium, the cells were stained with Hoechst 33342 for 10 min at 37 °C, then washed with PBS three times, and observed by LSCM (Ex: 488 nm and Em: 500–550 nm for YFP, while Ex: 405 nm and Em: 417‐477 nm for Hoechst 33342). The yellow fluorescence dots in the treated cells were counted using Image J.

### Cell Viability and Apoptosis Assay

To examine the cytotoxicity of LNPs, 143B cells were seeded in a 96‐well culture plate overnight until they reached 70% confluence, and then treated with LNPs at a dose of 64 µm in complete media for 24 h. After treatment, the cells were incubated with reagent of Cell Counting Kit‐8 (CCK‐8, Beyotime Biotechnology, Jiangsu, China), and cell viability was measured using a Multiskan GO microplate reader (Thermo Fisher Scientific Inc., Massachusetts, USA). For evaluating in vitro saporin delivery efficacy, 143B cells were incubated with free saporin or saporin/LNP at saporin concentrations ranging from 0 to 61 nm and a fixed LNP concentration of 64 µm for 24 h, followed by a CCK‐8 assay. For the apoptosis assay, 143B cells seeded in a 48‐well plate were incubated with saporin/LNP (3 nm saporin and 64 µm LNP) for 24 h. The cells were then washed with PBS and treated with Annexin V‐FITC and PI double‐staining apoptosis detection kit (Elabscience, Wuhan, China) according to the manufacturer's protocol. The percentage of apoptotic cells was determined by flow cytometry.

### Cas9 RNP Delivery

293T‐EGFP cells were seeded in a 24‐well plate overnight until they reached 40% confluence. The cells were incubated with Cas9/sgEGFP RNP‐loaded LNPs in complete medium (sequence of sgEGFP shown at Table S2, Supporting Information). The doses of Cas9 protein and sgEGFP in each well were 1.5 and 0.6 µg, respectively. The working concentration of LNPs was fixed at 32 µm. After 48 h, 293T‐EGFP cells were harvested and resuspended in 200 µL of PBS. Genomic DNA was isolated according to the protocols of FastPure Cell/Tissue DNA Isolation Mini Kit (Vazyme, Nanjing, China). The cell lysates were quantified and used as DNA templates to amplify the targeted genomic loci by PCR (primers listed in Table S3, Supporting Information). The samples were tested by Sanger sequencing (Saiheng Biotech., Shanghai, China), and the data were analyzed by the ICE CRISPR Analysis Tool (Synthego, California, USA).

### LNP Penetration in a 3D Tumor Spheroid Model

3D multicellular tumor spheroid model was used to evaluate the penetration capability of LNPs. Saporin was pre‐labeled with the near‐infrared fluorescent dye Cy5.5 (abbreviated as Cy5.5‐saporin) for 3D tumor spheroid imaging. 143B cells were diluted to 100 cells per microliter in complete medium containing 0.24% (w/v) methylcellulose. ≈2500 cells in 25 µL were pipetted and suspended inside a culture dish. After one day of culture, the droplets containing cell spheres were transferred to a 96‐well plate pre‐coating with 1% (w/v) agarose, and 100 µL of complete culture medium was added for a further 24 h incubation. Cell spheres of similar size were then cultured with Cy5.5‐saporin or Cy5.5‐saporin/LNP at different saporin concentrations for 16 h, followed by observation through confocal microscopy (Ex: 633 nm, Em: 650–700 nm) to evaluate the penetration depth of LNPs in the spheroids.

### In Vivo Saporin Delivery by LNPs

All the animal experiments were approved by Ethics Committee of East China Normal University (Approval number: ARXM2023002) and were conducted in accordance with NIH guidelines. Six‐week‐old female Balb/c mice (≈20 g) were injected subcutaneously with 143B cells (≈4 × 10^6^ cells in PBS) on the right back. When the 143B tumors grew to ≈80 mm^3^, the mice were randomly divided into four groups. Each group was intravenously injected with 100 µL PBS, LNP, saporin or saporin/LNP once every two days. Tumor size and body weight were recorded every two days. The doses of saporin and LNP in in vivo experiments were fixed at 50 µg kg^−1^ and 2.4 mg lipid/kg, respectively. After a total of five injections, the mice were sacrificed on day 10 to harvest the major organs. The harvested heart, liver, spleen, lung, and kidney were fixed at 4% formalin, embedded in paraffin, and sectioned into 4 µm slices. The sections were stained with hematoxylin and eosin and observed under an optical microscope.

To trace the in vivo biodistribution of LNPs, the LNPs were labeled with the hydrophobic near‐infrared dye DiR (designated as DiR‐LNP). One week after tumor inoculation with 143b cells, the tumor‐bearing mice were randomly divided into three groups and injected via tail vein with DiR, DiR‐LNP or Saporin/DiR‐LNP. After 24 h, the mice were euthanized, and tumors and organs were collected for imaging and fluorescence quantification using an in vivo imaging system (IVIS spectrum, PerkinElmer, Massachusetts, USA).

### IL‐10 Delivery by LNPs for Enhanced ACT in Mice Bearing B16F10 Tumor

B16F10‐OVA cells (10^6^ cells mouse^−1^) were injected subcutaneously into C57BL/6 mice. When the average tumor volumes reached 100 mm^3^, the mice received intravenous injections of activated OT‐1 CD8^+^ T cells (1.5 × 10^6^ cells mouse^−1^), followed by intravenous administration of PBS, LNP, IL‐10 or IL‐10/LNP (3 µg IL‐10/mouse, 48 µg LNP/mouse, every two days). Tumor size was measured using a caliper. After treatment, the tumors were excised and weighed at the indicated time. The collected tumors were placed into grinding tubes with pre‐cooled PBS (1 µL mg^−1^), and ground using a tissue grinder. The tumor tissue supernatant was taken after centrifugation at 12000 rpm for 5 min, and stored at −80 °C. IL‐10 levels in the tumor tissue supernatant were determined using mouse ELISA (MultiSciences, Hangzhou, China) according to the manufacturer's protocol. For blood IL‐10 levels, mouse blood samples were collected by orbital bleeding, clotted for 30 min at room temperature, and serum was taken after centrifugation at 3000 rpm for 15 min. Serum IL‐10 levels were determined using mouse ELISA, following the manufacturer's protocol.

### T Cell Infiltration Assay

B16F10‐OVA tumors were minced into small pieces (less than 3 mm in diameter) and resuspended in RPMI 1640 with 400 U mL^−1^ Collagenase IV (Gibco) and 30 U mL^−1^ DNase I (Gibco). The tumor pieces were incubated at 37 °C for half an hour, then incubated in complete medium (RPMI 1640 containing 10% FBS), and filtered through a 70 µm nylon cell strainer (Corning, New York, USA). After red blood cell lysis, tumor single‐cell suspensions were prepared for staining. All samples were blocked FcγII/III with anti‐CD16/32 (BD Pharmingen, BD, New Jersey, USA) at 4 °C for 30 min, and Fixable Viability Stain 780 (BD Pharmingen) was used to gate out non‐viable cells. Samples were then stained with CD3 and Vα2 fluorescence‐conjugated antibodies at 4 °C for 30 min. All samples were run on an LSRFortessa (BD Pharmingen) and analyzed using FlowJo software (BD, New Jersey, USA).

### Statistical Analysis

Statistical analyses were performed using Graphpad Prism 10.1.1 (Graphpad Software, California, USA). Except for the animal experiments, each group was tested in triplicate. Data were presented as mean ± standard deviation (SD), unless otherwise specified. One‐way ANOVA was used to compare means among three or more groups, while a *t*‐test was used for comparisons between two groups. Statistical significance was determined by *p*‐values, where *^N.S.^p* > 0.05 was considered no significant, ^*^p < 0.05 indicated a significant difference. ^**^p < 0.01 indicated a very significant difference, and ^***^p < 0.001 indicated an extremely significant difference.

## Conflict of Interest

The authors declare no conflict of interest.

## Supporting information



Supporting Information

## Data Availability

The data that support the findings of this study are available from the corresponding author upon reasonable request.
