# Supplementary material for: Optimization of Lipid Nanoparticles with Robust Efficiency for the Delivery of Protein Therapeutics to Augment Cancer Immunotherapy
Source: Adv Sci (Weinh). 2025 Mar 8;12(17):2500844. doi: 10.1002/advs.202500844 (PMC12061287; doi:10.1002/advs.202500844)
Supplement: Supplementary file 1 — Supporting Information [file ADVS-12-2500844-s001.docx]

Supporting Information

Optimization of lipid nanoparticles with robust efficiency for the delivery of protein therapeutics to augment cancer immunotherapy

Lanfang Ren, Zeda Zhao, Yuqing Chao, Panting Yu, Zhoufang Mei*, Bing Du*, Yiyun Cheng*


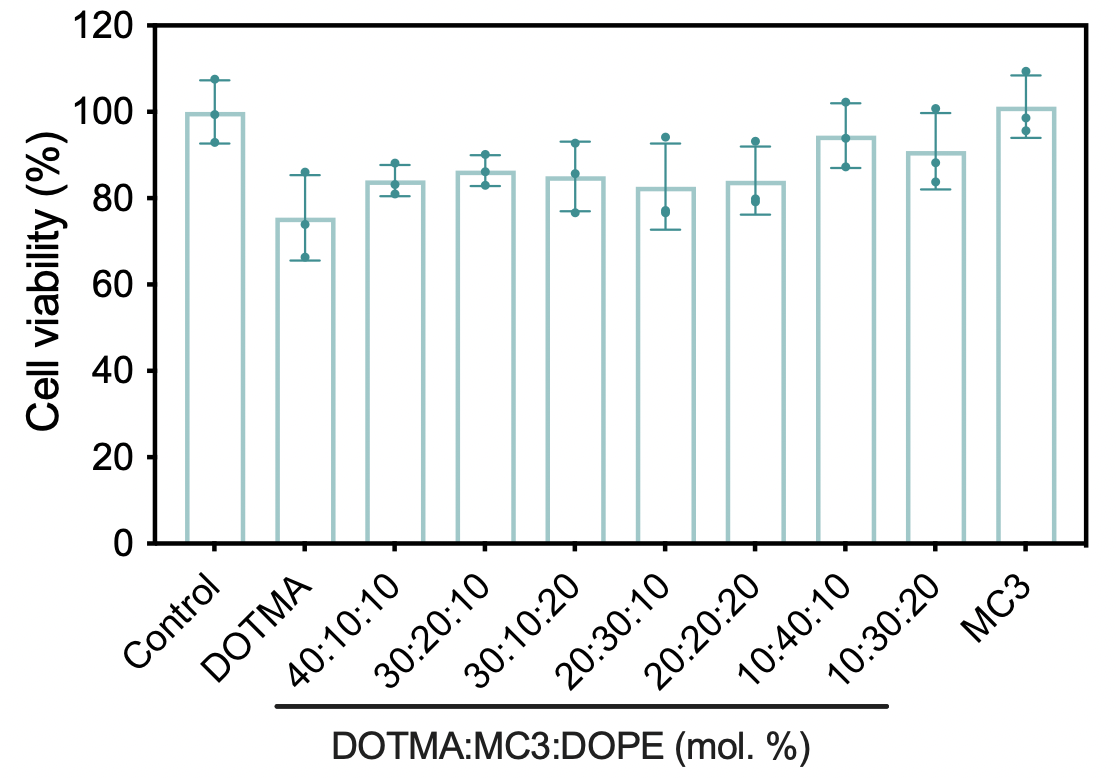
**
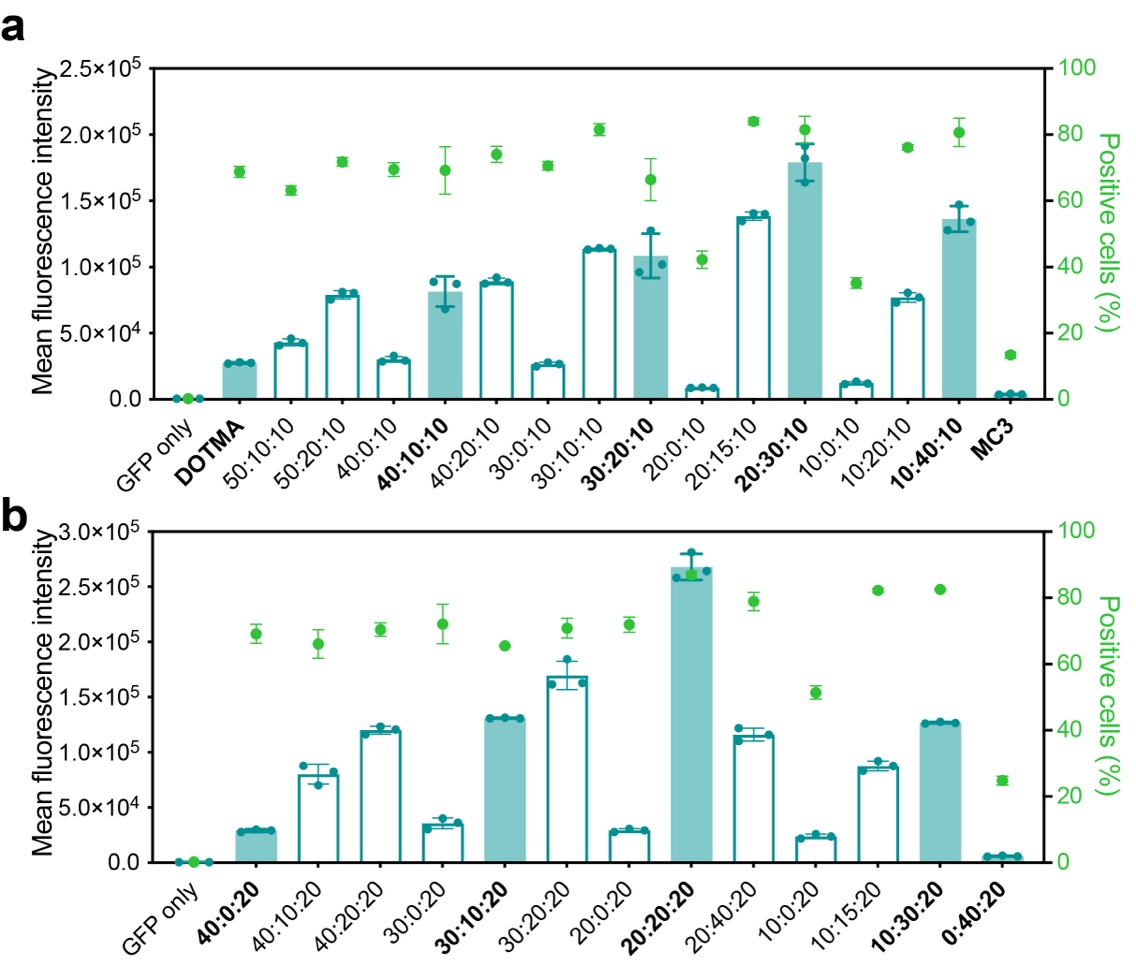
**Figure S1. Mean fluorescence intensity of 143B cells treated with LNPs prepared at a fixed DOPE/cholesterol/DMG-PEG molar ratio of (a) 10:38.5:1.5 or (b) 20:38.5:1.5. This experiment was conducted to evaluate the role of MC3 incorporation in cationic LNPs.

Figure S2. Viability of 143B cells treated with different LNPs.


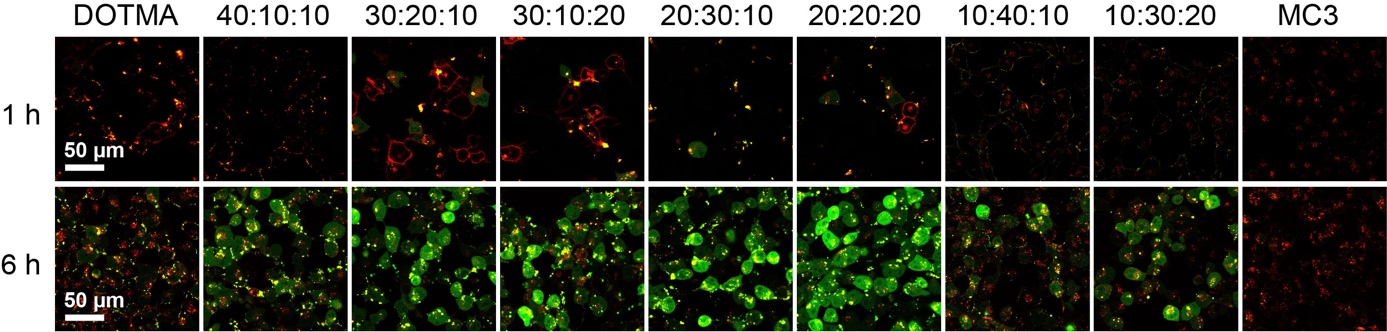
****Figure S3. Relative fluorescence intensity of 143B cells treated with LNPs (20:20:20) formulated in various buffers (pH 7.4).


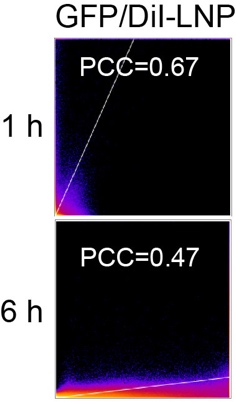
Figure S4. Confocal images of 143B cells treated with various LNP formulations loading with GFP for 1 hour or 6 hours. The LNPs were pre-labeled with DiI prior to treatment. GFP and DiI were visualized by green and red fluorescence, respectively.

Figure S5. Representative fluorescence colocalization analysis of 143b cells treated with the optimized LNP formulation (20:20:20, shown in Figure S4) at different time points.


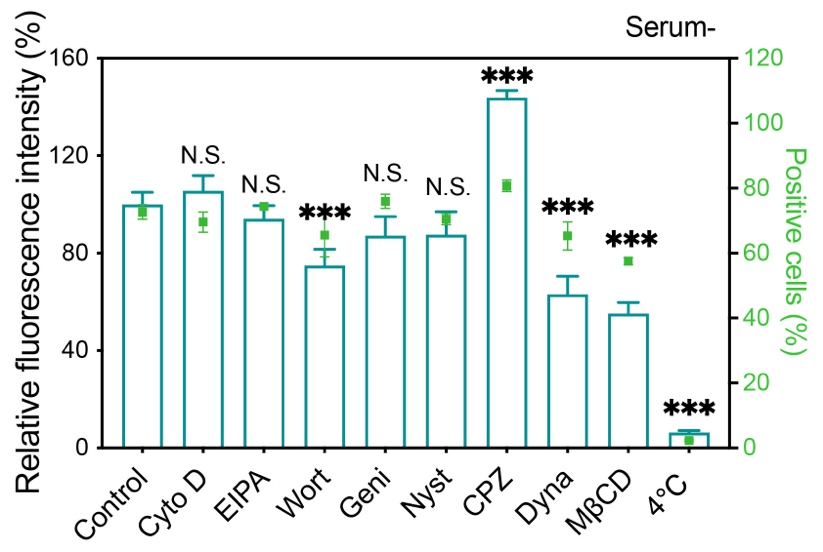

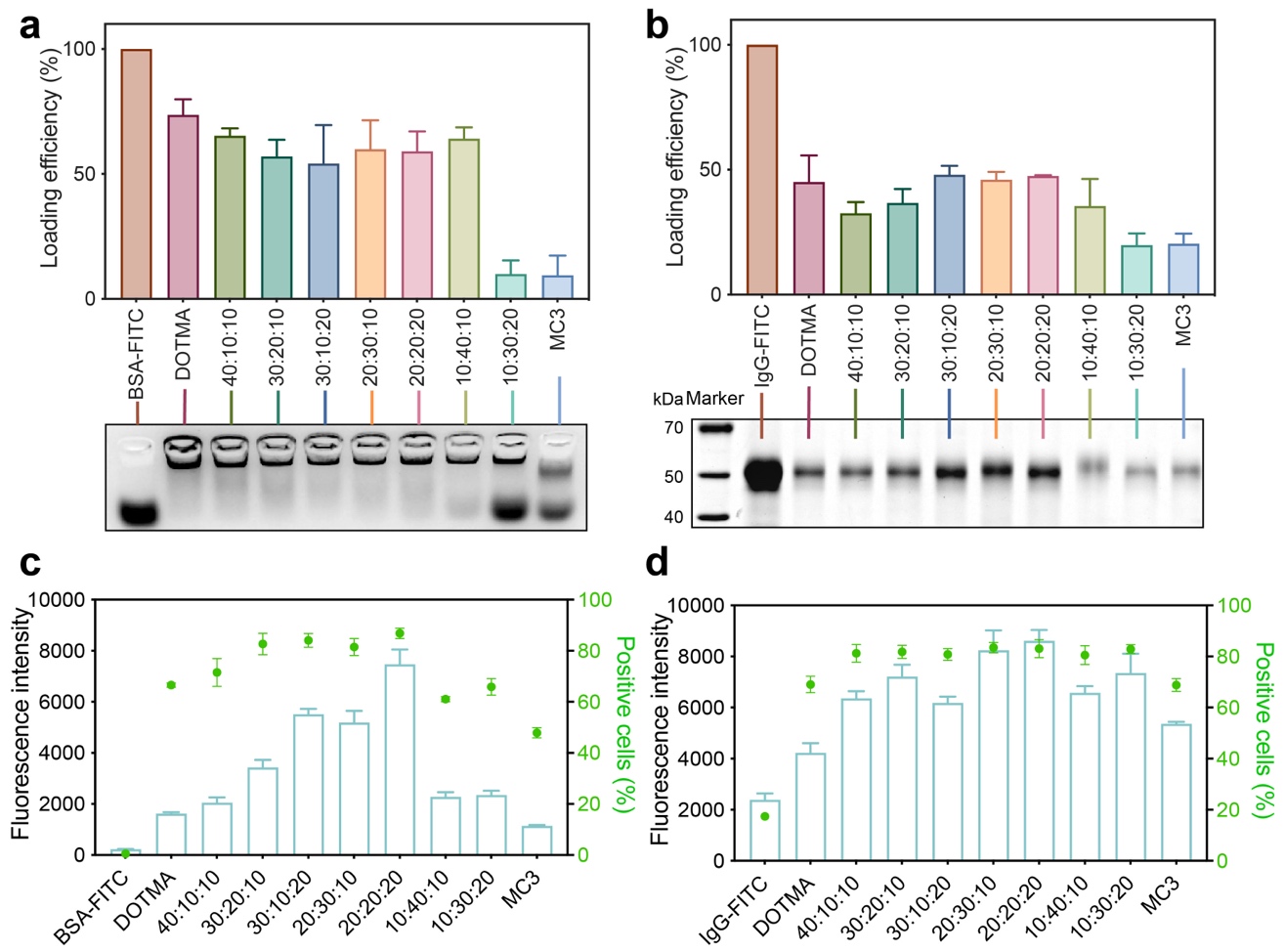
Figure S6. (a) Agarose gel electrophoresis of LNPs loading with BSA-FITC and (b) SDS-PAGE electrophoresis of LNPs loading with IgG-FITC. The loading ratio of BSA-FITC or IgG-FITC on LNPs was calculated according to the electrophoresis result (*n* = 3). Mean fluorescence intensity of 143B cells treated with LNP formulations loading with (c) BSA-FITC or (d) IgG-FITC.

Figure S7. Relative fluorescence intensity of LNP-treated cells pretreated with different endocytosis inhibitors in the absence of FBS.


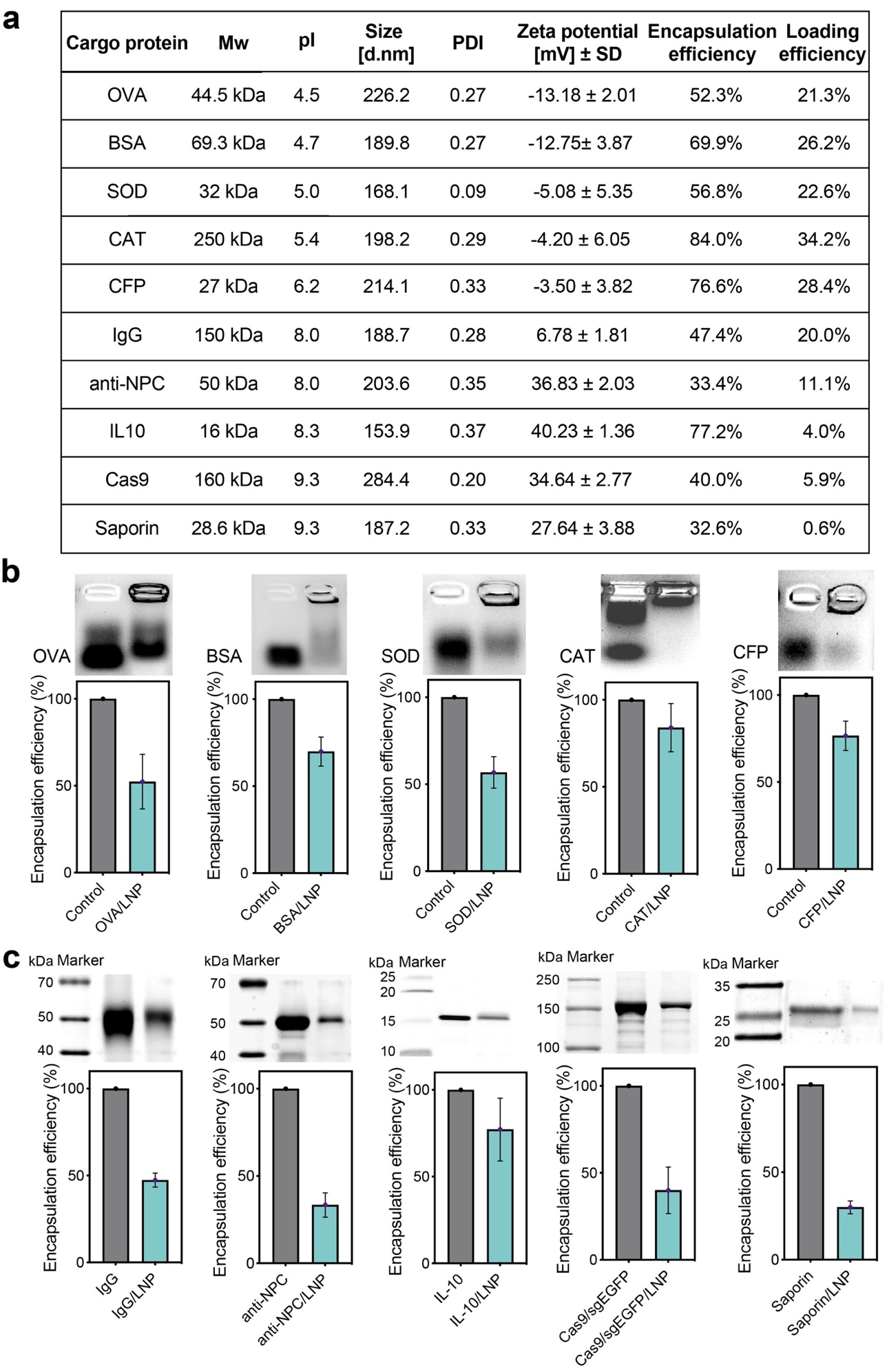
Figure S8. (a) Overview of cargo protein properties and characterization of LNPs encapsulating these proteins. (b) Agarose gel electrophoresis (for proteins with pI < 7.5) and (c) SDS-PAGE electrophoresis (for proteins with pI ≥ 7.5) were used to analyze the encapsulation efficiency of
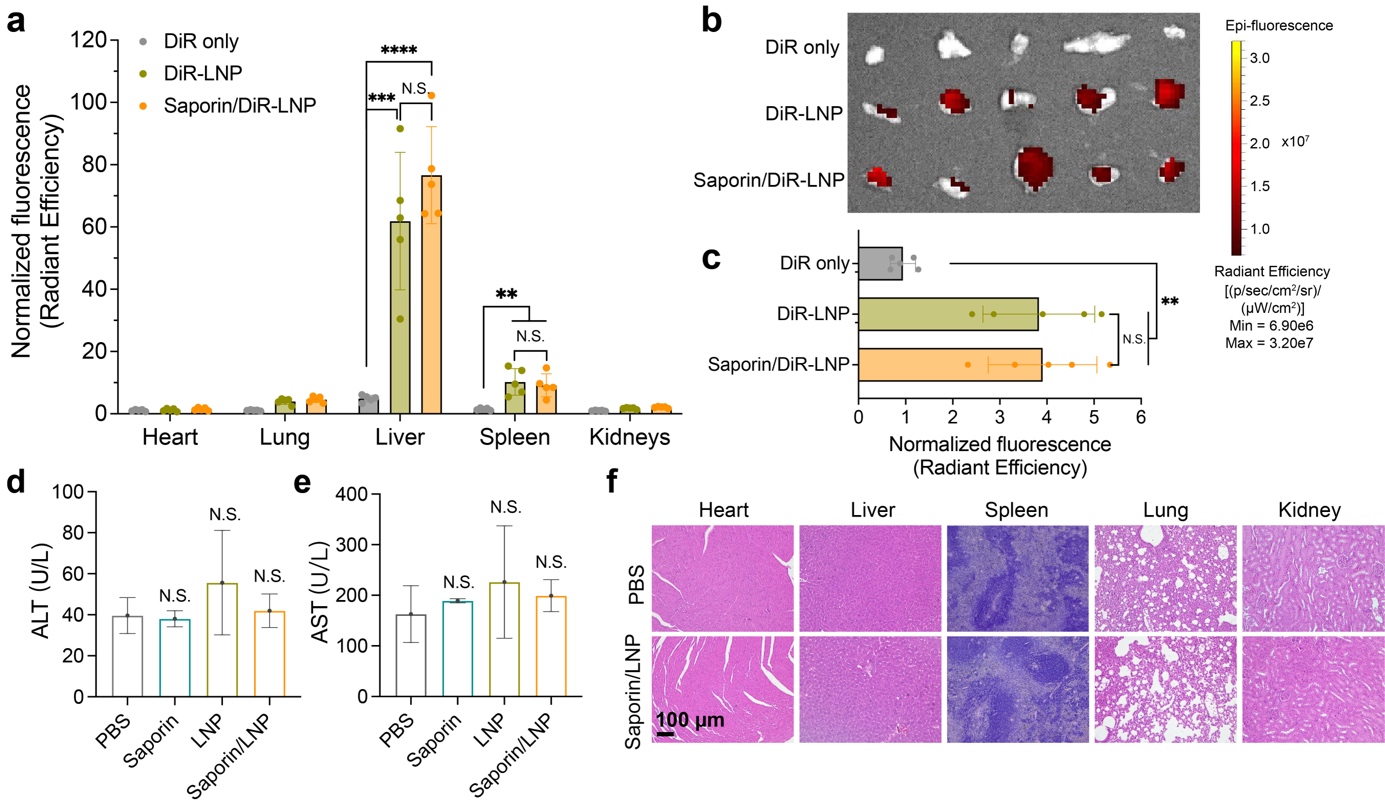
proteins. The total protein for each LNP encapsulation was used as a control.

Figure S9. (a) Quantitative analysis of the in vivo biodistribution of major organs following treatment of mice with DiR, DiR-LNP, or Saporin/DiR-LNP for 24 hours (*n* = 5). (b) IVIS imaging and (c) quantification of DiR fluorescence in tumors 24 hours after intravenous injection (*n* = 5). Fluorescence signals were normalized to the background before statistical analysis. (d, e) Serum ALT and AST levels in mice after 10 days of treatment. (f) Hematoxylin and eosin staining of major organ slices from mice treated with PBS or saporin/LNP on day 10.


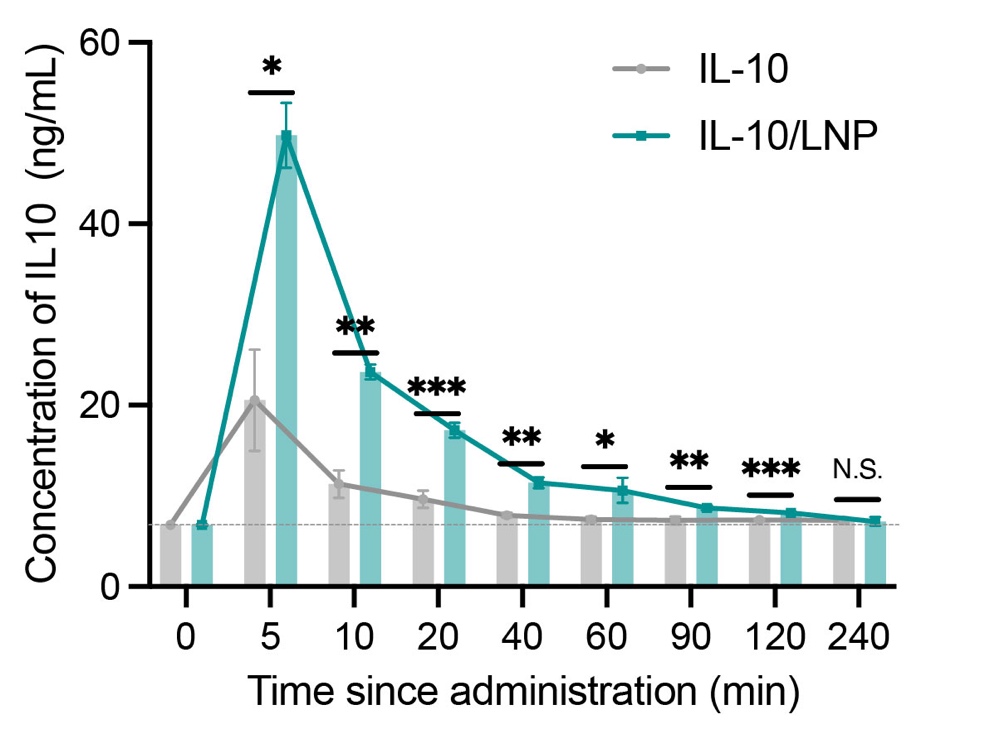


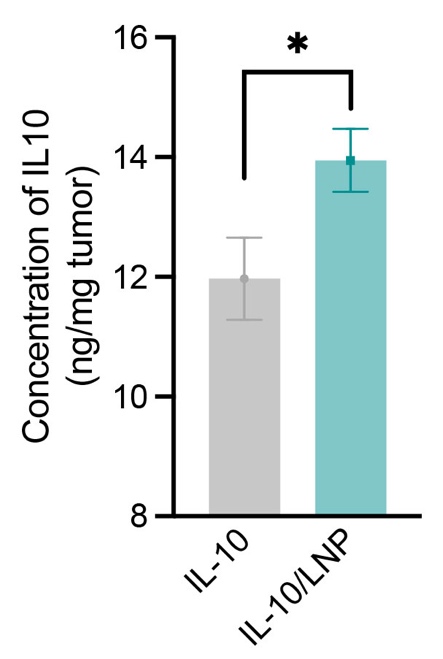
Figure S10. Concentrations of IL-10 in the blood collected from C57BL/6 mice administrated by IL-10 and IL-10/LNP, respectively (*n* = 3).


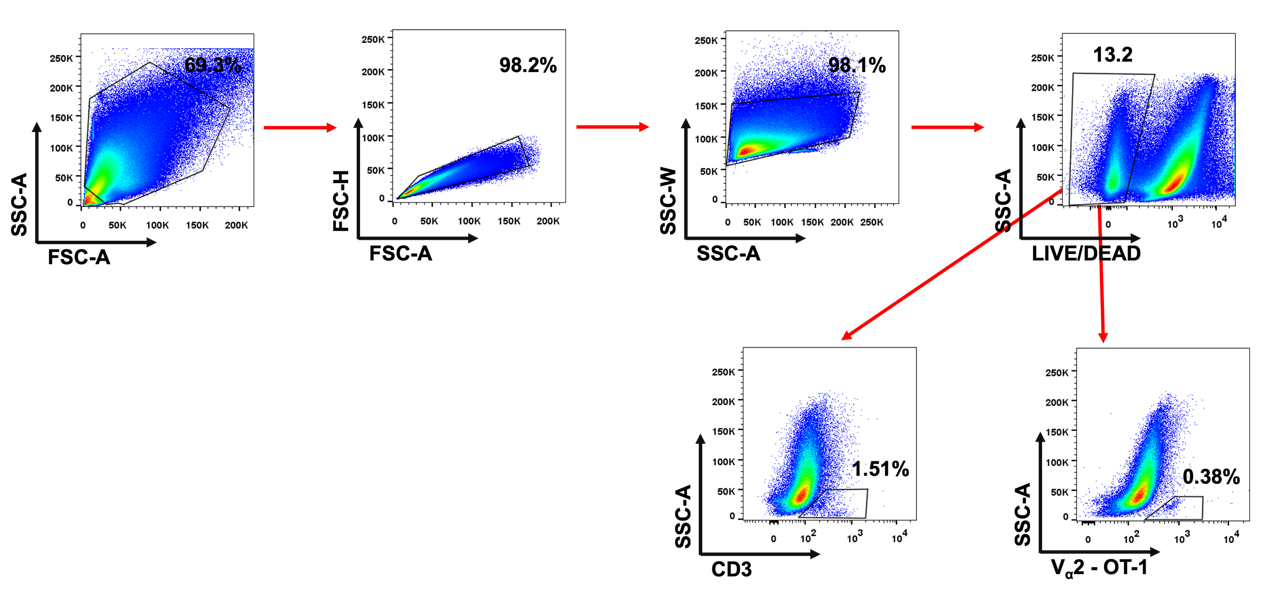
Figure S11. Concentration of IL-10 in the tumor tissues of C57BL/6 mice 4 hours after *i.v.* injections of IL-10 and IL-10/LNP, respectively.

Figure S12. Representative gating strategies of flow cytometry analysis for the quantification of tumor-infiltrating CD3^+^ and V_α_2^+^ OT-1 T cells in B16F10 tumors.

| **Abbreviation of LNPs** | **Molar ratio (mol. %)** | | | | | **Total lipid concentration (µM)** |
| --- | --- | --- | --- | --- | --- | --- |
|  | **DOTMA** | **MC3** | **DOPE** | **Cholesterol** | **DMG-PEG** |  |
| **DOTMA** | 50 | 0 | 10 | 38.5 | 1.5 | **64.00** |
| 50:10:10 | 50 | 10 | 10 | 38.5 | 1.5 | 70.40 |
| 50:20:10 | 50 | 20 | 10 | 38.5 | 1.5 | 76.80 |
| 40:0:10 | 40 | 0 | 10 | 38.5 | 1.5 | 57.60 |
| **40:10:10** | 40 | 10 | 10 | 38.5 | 1.5 | **64.00** |
| 40:20:10 | 40 | 20 | 10 | 38.5 | 1.5 | 70.40 |
| 30:0:10 | 30 | 0 | 10 | 38.5 | 1.5 | 51.20 |
| 30:10:10 | 30 | 10 | 10 | 38.5 | 1.5 | 57.60 |
| **30:20:10** | 30 | 20 | 10 | 38.5 | 1.5 | **64.00** |
| 20:0:10 | 20 | 0 | 10 | 38.5 | 1.5 | 44.80 |
| 20:15:10 | 20 | 15 | 10 | 38.5 | 1.5 | 54.40 |
| **20:30:10** | 20 | 30 | 10 | 38.5 | 1.5 | **64.00** |
| 10:0:10 | 10 | 0 | 10 | 38.5 | 1.5 | 38.40 |
| 10:20:10 | 10 | 20 | 10 | 38.5 | 1.5 | 51.20 |
| **10:40:10** | 10 | 40 | 10 | 38.5 | 1.5 | **64.00** |
| **MC3** | 0 | 50 | 10 | 38.5 | 1.5 | **64.00** |
| **40:0:20** | 40 | 0 | 20 | 38.5 | 1.5 | **64.00** |
| 40:10:20 | 40 | 10 | 20 | 38.5 | 1.5 | 70.40 |
| 40:20:20 | 40 | 20 | 20 | 38.5 | 1.5 | 76.80 |
| 30:0:20 | 30 | 0 | 20 | 38.5 | 1.5 | 57.60 |
| **30:10:20** | 30 | 10 | 20 | 38.5 | 1.5 | **64.00** |
| 30:20:20 | 30 | 20 | 20 | 38.5 | 1.5 | 70.40 |
| 20:0:20 | 20 | 0 | 20 | 38.5 | 1.5 | 51.20 |
| **20:20:20** | 20 | 20 | 20 | 38.5 | 1.5 | **64.00** |
| 20:40:20 | 20 | 40 | 20 | 38.5 | 1.5 | 76.80 |
| 10:0:20 | 10 | 0 | 20 | 38.5 | 1.5 | 44.80 |
| 10:15:20 | 10 | 15 | 20 | 38.5 | 1.5 | 54.40 |
| **10:30:20** | 10 | 30 | 20 | 38.5 | 1.5 | **64.00** |
| **0:40:20** | 0 | 40 | 20 | 38.5 | 1.5 | **64.00** |

Table S1. LNPs with different formulations and working concentrations were abbreviated according to the molar ratio of DOTMA/MC3/DOPE. Formulations of 50:0:10 and 0:50:10, indicating no MC3 or no DOTMA involved in the LNPs, were abbreviated as DOTMA and MC3, respectively. The molar ratio of cholesterol and DMG-PEG in all the LNPs was fixed at 38.5% and 1.5%, respectively.

| **Name** | **Target sequences (5’ to 3’)** |
| --- | --- |
| sgEGFP | GAAATTAATACGACTCACTATAGGGGGCACGGGCAGCTTGCCGGGTTTTAGAGCTAGAAATAGC |

Table S2. The sequence of sgEGFP in this study.

| **Name** | **Forward primers** | **Reverse primers** |
| --- | --- | --- |
| EGFP | ATGGTGAGCAAGGGCGAG | TTACTTGTACAGCTCGTCCATGC |

Table S3. Primers for PCR amplification of target gene in this study.
